# Laser-Assisted Patterning of Metallized Fabrics for Washable and Durable Electronic Textiles

**DOI:** 10.1007/s42765-026-00761-8

**Published:** 2026-08-24

**Authors:** Muhammad Masud Rana, Sotoudeh Sedaghat, Akshay Krishnakumar, Devendra Sarnaik, Praveen Srinivasan, Yashwanth Ramesh, Md Mahabubur Rahman, Rahim Rahimi

**Affiliations:** 1 School of Electrical and Computer Engineering, Purdue University, West Lafayette, IN 47907 USA; 2 Birck Nanotechnology Center, Purdue University, West Lafayette, IN 47907 USA; 3 School of Materials Engineering, Purdue University, West Lafayette, IN 47907 USA

**Keywords:** Laser-assisted patterning, Wearable electronics, Electrical impedance tomography, Wearable health monitoring, Metallized fabrics

## Abstract

Wearable electronic textiles (e-textiles) are increasingly being explored for continuous, noninvasive physiological monitoring; however, scalable fabrication of high-resolution circuitry with long-term mechanical and washing durability remains a major challenge. Conventional approaches, including screen printing, inkjet printing, and conductive-thread embroidery, enable the integration of conductive traces into garments but often suffer from limited patterning resolution, binder-related degradation, mechanical instability, and unreliable interfacing with electronic components. Here, we present a selective laser-assisted ablation method for directly defining conductive circuit traces on single-sided metallized fabrics (MFs) composed of woven, multi-stranded silver fiber yarns. This mask-free and chemical-free process enables rapid, digitally programmable patterning while preserving the structural integrity, flexibility, and breathability of the underlying textile. Importantly, localized laser heating simultaneously removes conductive fibers in selected regions and thermally fuses the exposed ends of adjacent silver yarns, thereby suppressing fraying and enhancing the mechanical robustness of the patterned circuit edges. Using a neodymium-doped yttrium aluminum garnet (Nd:YAG) laser tuned for high absorption by silver, controlled ablation at energy densities of 8–26.5 J·cm^−2^ produced well-defined insulating regions without compromising the conductivity of neighboring traces. Surface and elemental analyses confirmed nearly complete silver removal, with residual silver content below 3% in the ablated areas. The resulting laser-ablated metallized fabric (LA-MF) exhibited low sheet resistance (approximately 2 mΩ/sq.), retained more than 94% of its initial conductivity after 10000 bending cycles, and maintained stable electrical performance after more than 20 machine-washing cycles. As a proof of concept, a 16-electrode electrical impedance tomography (EIT) array was patterned directly onto the MF and integrated into a compression garment for noninvasive lung-function monitoring. The textile electrodes enabled real-time impedance imaging, and an embedded convolutional neural network accurately classified respiratory states, including normal breathing and breath-hold conditions, even after repeated laundering. This laser-assisted patterning framework provides a robust, scalable, and manufacturing-compatible route for integrating high-performance circuit architectures directly into fabrics for wearable sensing, healthcare monitoring, and next-generation smart textile applications.

**Graphical Abstract:**

**Figure:**
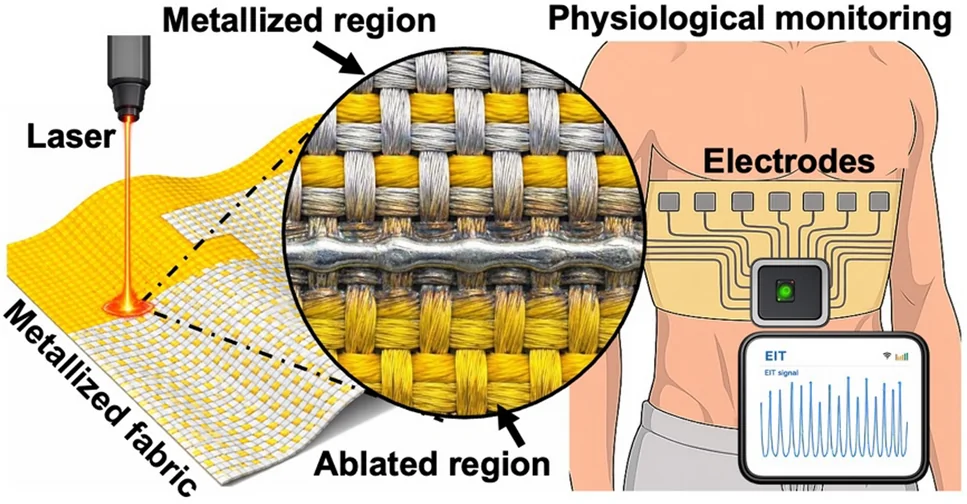

**Supplementary Information:**

The online version contains supplementary material available at https://doi.org/10.1007/s42765-026-00761-8.

## Introduction

Integrated electronics have transformed both daily life and modern healthcare by progressively shrinking sensing, computation, storage, and wireless communication into personal devices that can operate continuously at the point of use [1]. What began with the miniaturization that enabled portable consumer electronics has evolved into body-adjacent technologies, such as smartwatches, fitness bands, smart glasses, patches, and other wearable sensor platforms [2, 3]. The scale of this transition is substantial: mobile technologies and services were estimated to contribute about $6.5 trillion, or 5.8% of global GDP, while worldwide wearable-device shipments reached 611.5 million units in 2025 [4, 5]. Within that broader market, wrist-worn devices alone shipped 45.6 million units in the first quarter of 2025, including 34.8 million smartwatches [6]. Beyond their commercial success, these systems have changed how health-related information is acquired by enabling real-time and increasingly continuous measurement of physiological and biochemical signals outside conventional episodic clinical assessment [7, 8]. Wearable sensors are now capable of mining health information in a noninvasive or minimally invasive manner across watches, patches, masks, jewels, and textiles, thereby supporting higher-resolution and longer-duration observation of human physiology in everyday environments [9, 10].

The next major step in this progression is to move electronics from discrete accessories into the garments people already wear every day. Although wrist-worn systems, head-mounted devices, and skin-mounted patches have demonstrated the value of continuous monitoring, many of these platforms remain constrained by rigid or semi-rigid architectures and by their limited ability to establish intimate, stable, and spatially distributed contact across large and anatomically diverse body surfaces [9, 11, 12]. Textiles, by contrast, offer softness, breathability, conformability, moisture management, and broad body-area coverage that are difficult to reproduce with conventional thin-film substrates [13–15]. Porous conductive textiles have therefore emerged as especially attractive material platforms for wearable electronics, because they combine electrical functionality with the flexibility, permeability, and wearing comfort expected of conventional garments [16]. For long-duration monitoring, these attributes are not peripheral; they are central to whether a system can be worn naturally and used reliably in real settings [16].

This vision has driven rapid growth in electronic textiles, or e-textiles, in which sensing and electronic functionality are incorporated directly into fibers, yarns, fabrics, or garment assemblies [17, 18]. Textile-based systems have already been explored for electrophysiological monitoring of signals, such as electrocardiography, electromyography, and electroencephalography; for biomechanical and physical monitoring of respiration, pressure, temperature, posture, gait, and sleep; and for biochemical analysis of sweat-borne markers including pH, sodium, uric acid, glucose, lactate, and cortisol [19, 20]. Such capabilities are particularly attractive in contexts where comfort, body coverage, and continuous use matter most, including daily wellness tracking, rehabilitation, and long-term biomedical monitoring [21, 22]. Systematic reviews of biomedical smart clothing further emphasize that the comfort, ease of use, and ubiquity of garments may be decisive advantages for sustained clinical deployment, precisely because they lower the barrier between sensing technology and ordinary wear [23].

To realize that promise, however, garments must do more than host isolated sensors. They must also contain distributed electrical interconnects that can route signals and power across the textile to measurement sites, connectors, and readout electronics [24, 25]. In practice, this requires circuit traces that simultaneously deliver high electrical conductivity, low mechanical impedance, washability, breathability, and a tactile character consistent with everyday fabrics [25, 26]. Recent reviews of textile electronics identify this integration problem as one of the major barriers to practical systems: textile devices require reliable conductive pathways and connectors, yet the final garment must still remain flexible, lightweight, stretchable, washable, breathable, and unobtrusive [27, 28]. The central challenge in e-textiles is therefore not only the realization of sensors, but also the creation of textile-native circuitry that preserves garment utility while approaching the electrical performance expected from conventional electronics [18, 28].

To date, three principal strategies have dominated the fabrication of textile interconnects [28–30]. The first is based on printing technologies, particularly screen printing and inkjet printing, which adapt conventional graphic and textile manufacturing methods to deposit conductive patterns onto fabric surfaces [31, 32]. In these approaches, a conductive ink or paste containing metallic nano- or microparticles is deposited onto the textile and subsequently dried or cured [32, 33]. Polymeric binders are typically used to immobilize the particles on the rough, porous substrate and to maintain conductive continuity after drying, so charge transport occurs through a percolative network formed between adjacent particles [32, 34]. This route is attractive, because it is low-profile, design-flexible, and compatible with well-established large-area printing infrastructure [35]. It also allows conductive features to be added after textile manufacture, which makes it appealing for rapid prototyping and low-temperature processing of sensitive substrates [33, 36].

Despite these advantages, printed textile traces remain limited by the particulate nature of the conductive pathway and by the chemistry required to maintain it. Recent reviews identify oxidative degradation and limited shelf life as persistent problems for metallic inks, especially nanoparticle-based systems, while highly conductive formulations based on silver nanoparticles remain relatively expensive and are not generally known for withstanding the severe washing and wearing conditions expected in garments [37, 38]. Even when printing is successful, practical feature fidelity on textiles is often coarse, because substrate roughness, pore structure, wetting, and ink penetration distort the intended geometry [31]. For example, a recent screen-printing study using conductive pastes on flexible substrates reported that a nominal 1 mm mask produced silver-chloride lines about 1.24 mm wide and carbon lines about 1.36 mm wide [39]. Likewise, a recent washable inkjet study on polyester fabric reported conductive lines in the approximate width range of 209–325 µm, underscoring that the textile substrate still constrains fine-feature reliability even in advanced formulations [38]. In addition, screen-printed textile interconnects often show limited wash and bending durability and frequently require smoothing or encapsulation layers, which adds complexity and can compromise the native tactile and transport properties of the textile [40, 41].

A second widely used strategy relies on conductive yarns, metal filaments, or plated fibers integrated through embroidery, weaving, knitting, or related textile processes. These approaches are attractive, because the conductive elements are introduced through native textile manufacturing routes and therefore preserve, to a greater extent, the softness and deformability of the host fabric [42, 43]. Conductive yarns embedded by weaving, knitting, or embroidery are widely regarded as elegant and unobtrusive candidates for textile circuitry, because they integrate without fundamentally disturbing the textile nature of the system [44]. They also provide more continuous conduction than particle-based inks and can support longer signal paths with electrical behavior closer to bulk-metal transport [45]. However, failure and connection reliability remain major concerns. Studies on conducting yarns under mechanical loading show that bending, shearing, and tension can progressively damage conductive filaments or delaminate metallic coatings, producing unstable resistance and conductivity loss [27, 46]. At the system level, joining technologies remain a persistent problem, and reviews of e-textile connectors emphasize that relatively few solutions have been developed specifically for textile applications. Resolution is also often limited in practical embroidered geometries: earlier embroidery processes using thicker threads reported geometrical accuracy worse than 1 mm, and more recent embroidered structures still commonly exhibit line widths on the order of about 1 mm for linear stitches and roughly 1–5 mm for curved patterns [47, 48]. Although highly optimized embroidery demonstrations can push well below the millimeter scale, those specialized cases do not resolve the broader difficulties of fraying, soft-to-rigid interfacing, component attachment, and long-term durability under flexing and laundering [49].

A third strategy builds on flexible printed-circuit and microelectronic manufacturing by fabricating circuitry first and then attaching, laminating, or embedding it into the textile. This hybrid route inherits the major strengths of established circuit-board technology, including high routing density, well-defined trace geometry, and conductivity close to that of continuous metals [50, 51]. Encapsulated filament circuits integrated within textiles, for example, have been reported to survive 45 washing cycles and more than 1500 cycles of 90° bending [52]. More advanced direct textile microfabrication has also recently achieved sub-100 µm metal patterning while retaining permeability and washability [50]. Yet these advantages come with significant trade-offs. Flexible thin-film electronics generally exhibit poorer air and moisture permeability than textiles, which can reduce wearing comfort during long-term use, while direct in-textile photolithography still depends on multistep metallization, resist processing, exposure, development, and etching sequences that remain costly and fabrication-intensive [53–55]. More broadly, reviews of textile-integrated microelectronics note that textile-adapted and textile-integrated approaches still struggle to fully satisfy the simultaneous requirements of flexibility, washability, breathability, and unobtrusiveness needed for practical garments.

A practical route forward is therefore to combine the native feel and permeability of textiles with a circuit-definition method that approaches the precision and conductivity of conventional electronics without inheriting the full cost and complexity of photolithography or the electrical limitations of percolative inks [16]. Selective laser processing offers a particularly attractive pathway for this purpose. Laser–matter interactions are inherently wavelength-dependent, and the absorptivity of metals varies with both wavelength and metal type; accordingly, dissimilar material stacks composed of metals and polymers can exhibit strongly different coupling to the same laser beam [56, 57]. Recent reviews further note that pulsed Nd:YAG lasers operating near 1 µm are especially well suited to thin-metal processing because of their efficient coupling and reduced heat-affected zones relative to longer-wave systems [58, 59]. In textile substrates, laser cutting and engraving also provide an important mechanical benefit: synthetic fibers can locally melt at the cut edge, which suppresses unraveling and fraying [60]. These characteristics make selective laser subtraction especially relevant for metallized fibrous substrates, where a conductive surface must be removed with high spatial selectivity while preserving the underlying textile architecture [61, 62].

This concept is strongly supported by prior work on metallized paper. In a foundational comparison of direct and indirect laser ablation of metallized paper, direct Nd:YAG processing at 1.06 µm selectively removed the conductive aluminum coating without damaging the natural fibrous structure of the paper, whereas indirect CO_2_ processing at 10.6 µm achieved metal removal only through partial substrate etching [56, 63, 64]. Follow-on work then demonstrated one-step laser-induced patterning and sensor formation directly on metallized parchment paper, establishing Nd:YAG processing as a low-cost and scalable manufacturing route for paper-based circuitry and sensing [56, 65]. Building on that foundation, metallized fabrics (MFs) offer a compelling next substrate class. Driven by increasing demand for electromagnetic interference and radio-frequency shielding, conductive fabrics based on metal-coated textiles, metal wires, and conductive yarns are now widely manufactured in woven and related textile architectures [66, 67]. These materials are already designed to combine conductivity with flexibility, drape, and light weight, and therefore provide a ready-made starting platform for circuit fabrication if conductive regions can be selectively removed without destroying the textile scaffold [16, 68].

**Fig. 1:**
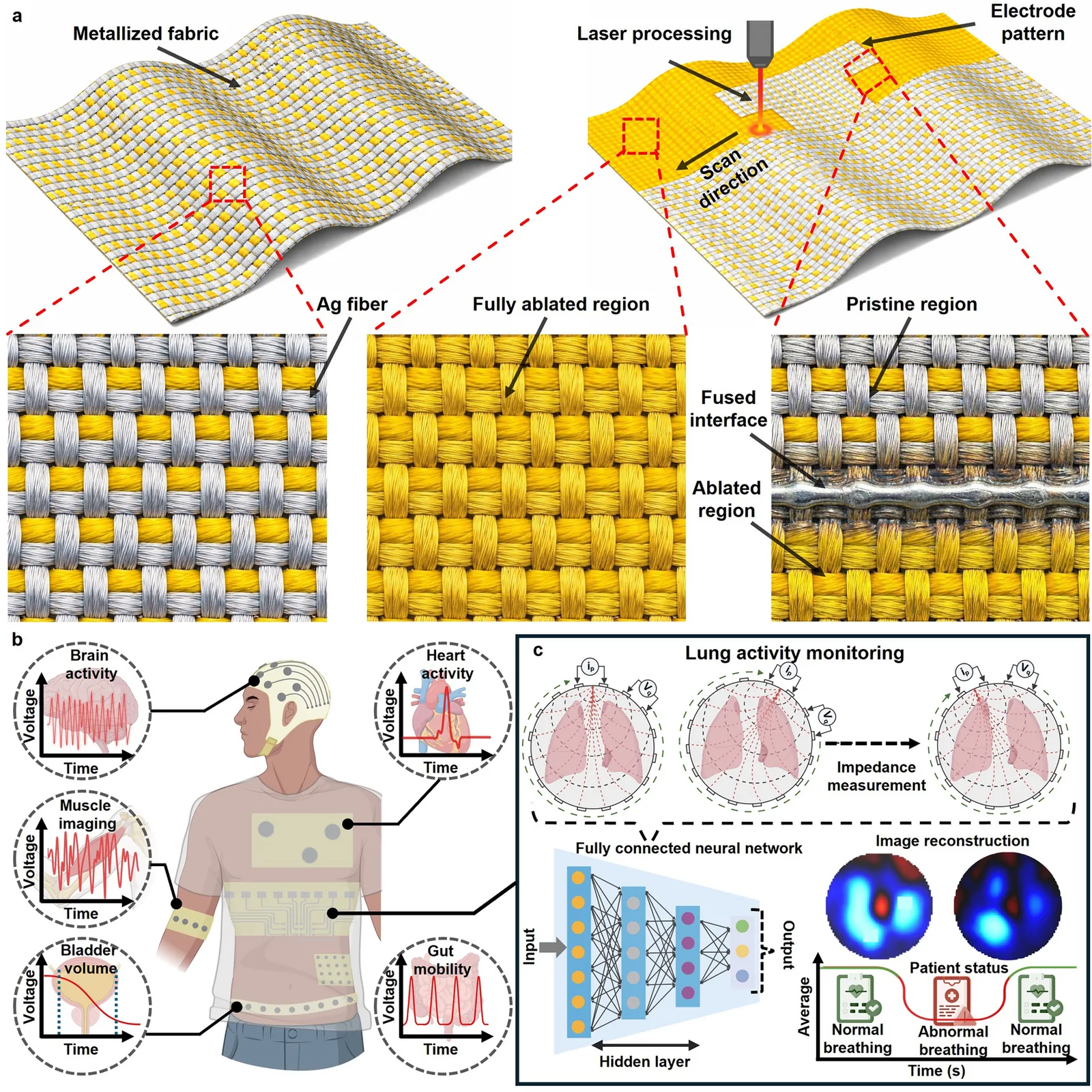
Schematic illustration of laser-assisted patterning of MFs and their integration into wearable electronic textiles for physiological monitoring. **a** Selective Nd:YAG laser ablation of woven silver fibers from a single-sided MF. Localized absorption by the conductive silver yarns enables precise vaporization of selected regions while simultaneously fusing adjacent fiber ends, suppressing fraying and producing sharply defined insulating domains without damaging the underlying polymer textile. **b** Digitally programmable fabrication of high-density conductive circuit traces directly on fabric for integration into wearable garments and other e-textile platforms. **c** Proof-of-concept application in a garment-integrated electrical impedance tomography system, where spatially distributed textile electrodes acquire impedance signals for tomographic reconstruction and neural-network-based classification of respiratory activity

In this work, we introduce a solvent-free, mask-free Nd:YAG laser-selective patterning strategy for MFs composed of woven conductive silver fibers and repurpose a commercially available shielding textile as a circuit-grade e-textile substrate (Fig. 1a). A computer-controlled laser is used to subtractively remove unwanted conductive regions and thereby define intricate electrical traces directly on the fabric while preserving the underlying textile architecture. By tailoring the laser-processing window, the method yields flexible, breathable, and highly conductive circuit pathways while the localized thermal interaction at cut boundaries suppresses fraying and helps preserve mechanical integrity at the patterned edges (Fig. 1b). As a proof of concept, this platform is used to fabricate a garment-integrated 16-electrode textile array for electrical impedance tomography of respiratory function, demonstrating dense routing, long conductive pathways, reliable interfacing with external electronics, and stable operation after repeated washing (Fig. 1c). Collectively, this work establishes selective laser patterning of MFs as a scalable route that bridges the gap between low-conductivity printed traces and comfort-limited laminated electronics, thereby advancing e-textiles toward practical, application-ready wearable systems.

## Results

### Optimization of Laser Processing Parameters

To enable the fabrication of practical and high-performance circuit traces, a systematic study was conducted to identify laser processing conditions that allow selective removal of conductive silver fibers from the MF while preserving the structural integrity of the underlying textile substrate. Achieving this balance is critical for defining conductive pathways with high precision without compromising the mechanical and functional properties of the fabric. A parametric study was performed by systematically varying laser power and scanning speed, thereby generating a range of laser energy densities (J·cm^−2^). These parameters were organized into a 10 × 10 processing matrix (totaling 100 distinct conditions), enabling comprehensive mapping of the laser–material interaction space. Laser processing was performed along both warp and weft directions to evaluate any directional dependence arising from the anisotropic woven architecture.

**Fig. 2:**
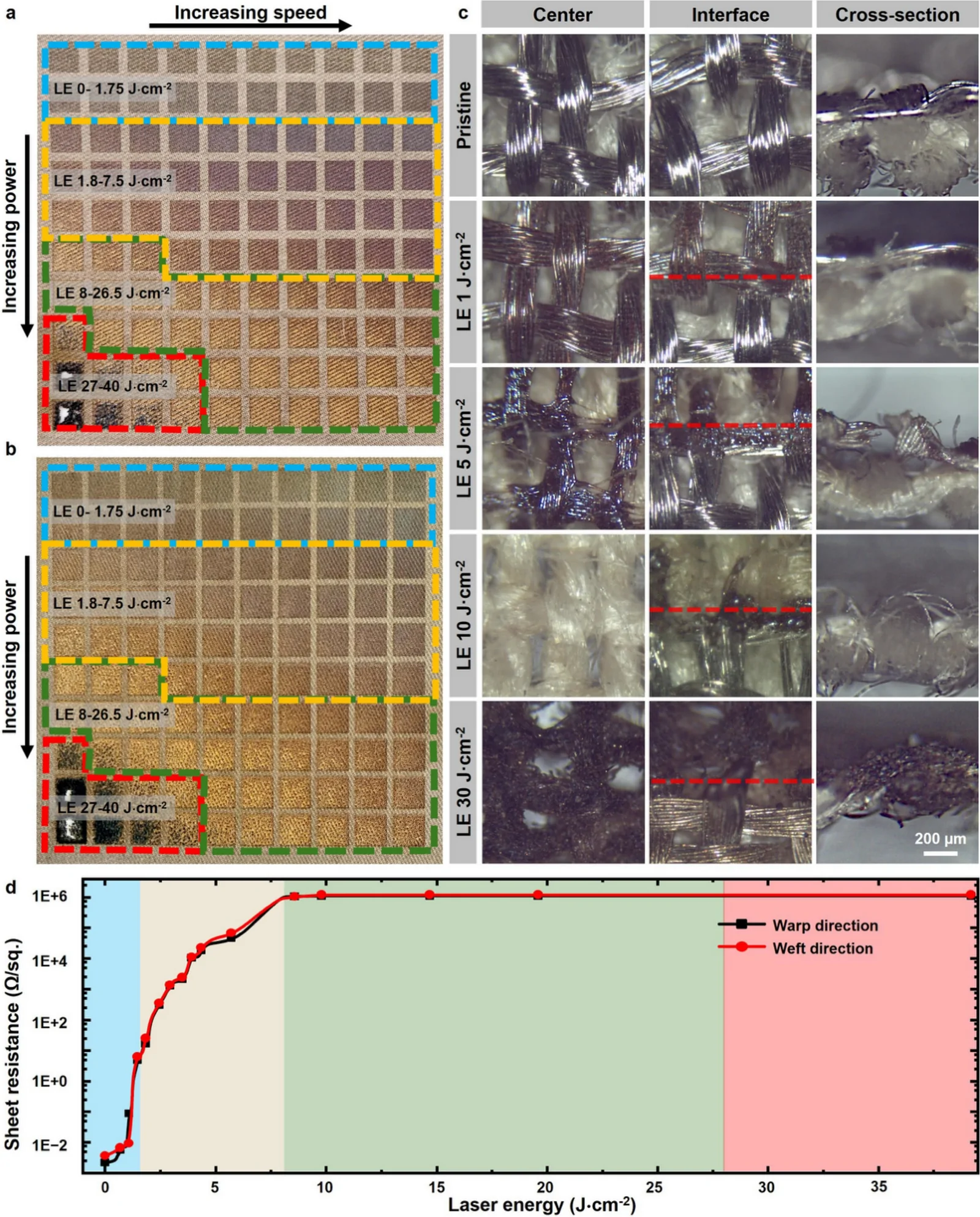
Optimization of selective laser patterning of MF and corresponding microstructural and electrical evolution. Process map generated from a 10 × 10 matrix of laser power and scanning speed combinations, illustrating four distinct laser-ablation regimes for MF patterned along the **a** warp and **b** weft directions: no observable modification (blue), partial metal removal (yellow), complete selective ablation (green), and thermally damaged textile (red). **c** Optical micrographs of the center region, ablation interface, and cross-section of pristine MF and representative samples processed at laser energy densities of 1, 5, 10, and 30 J·cm^−2^, showing the transition from intact silver yarns to partial melting, complete removal of conductive fibers, and substrate degradation. The dotted red line indicates the boundary between laser-ablated and unprocessed regions. **d** Sheet resistance of MF as a function of laser energy density for samples patterned along the warp and weft directions, identifying the optimized nonconductive processing window (8–26.5 J·cm^−2^) for selective removal of the conductive silver layer while preserving the underlying textile structure

Analysis of the processed samples revealed four distinct laser–material interaction regimes based on the applied energy density, as summarized in Fig. 2a, b. At low-energy densities (0–1.75 J·cm^−2^, blue region, *n* = 20), no significant modification of the metallized surface was observed, indicating sub-threshold conditions insufficient to induce melting or ablation of the silver fibers. At intermediate energy densities (1.8–7.5 J·cm^−2^, yellow region, n = 37), partial thermal interaction was observed, characterized by localized melting and deformation of the silver fibers without complete removal. In this regime, residual metallic fragments remained adhered to the textile surface, resulting in incomplete electrical isolation. At higher energy densities (8–26.5 J·cm^−2^, green region, n = 34), efficient and selective ablation of the conductive silver fibers was achieved. Within this regime, the laser energy was sufficient to fully remove the metallic layer, exposing the underlying polyester fibers while maintaining their structural integrity. In contrast, at excessive energy densities (27–40 J·cm^−2^, red region, n = 9), the laser-induced thermal load exceeded the tolerance of the textile substrate, resulting in fiber degradation, charring, and partial collapse of the woven structure. These observations establish a well-defined processing window for selective and damage-minimized laser patterning. Notably, similar processing trends were observed for both warp- and weft-oriented laser scans, indicating that the ablation behavior is largely independent of fiber orientation despite the inherent anisotropy of the woven structure. To further elucidate the structural evolution across these regimes, representative samples were selected for detailed microscopic analysis at energy densities of 1 J·cm^−2^ (low-energy), 5 J·cm^−2^ (intermediate), 10 J·cm^−2^ (optimal), and 30 J·cm^−2^ (high-energy), along with pristine MF. Optical microscopy was performed in top-view, edge-view (interface), and cross-sectional configurations, as shown in Fig. 2c.

In the pristine MF, all imaging modalities reveal continuous, highly reflective silver yarns with smooth surfaces and uniform filament bundling, tightly interwoven within the textile matrix. Cross-sectional imaging confirms that the conductive silver fibers are confined to the top surface layer, while the underlying polyester fibers form a uniform insulating structure. The polyester fibers exhibit diameters of approximately 7–10 µm, bundled into yarns of approximately 190–220 µm diameter, with consistent interlacing in both warp and weft directions. At low-energy density (1 J·cm^−2^), the processed samples exhibit minimal deviation from the pristine morphology. Top-view images show slightly reduced surface reflectivity and minor discoloration, suggesting limited thermal interaction. However, no evidence of material removal or structural disruption is observed. Cross-sectional images confirm the absence of melting or deformation in both the silver and polyester fibers, indicating that this regime corresponds to sub-threshold laser exposure. At intermediate energy density (5 J·cm^−2^), clear signs of thermal interaction with the silver fibers are observed. The silver filaments exhibit localized melting, coalescence, and partial fragmentation, leading to irregular and non-uniform surface morphology. Residual metallic material is frequently observed adhered to the underlying polyester fibers, indicating incomplete ablation. Interface regions display discontinuous separation of conductive pathways, while cross-sectional imaging reveals localized redeposition of molten silver onto the textile substrate. These features suggest insufficient energy for complete removal of the conductive layer.

In contrast, at an optimal energy density of 10 J·cm^−2^, the laser processing results in clean, spatially confined ablation of the silver fibers. Top-view images show complete removal of the conductive layer within the irradiated regions, revealing the underlying polyester fibers with high uniformity. Interface regions exhibit sharply defined boundaries between ablated and non-ablated areas, indicating precise control over pattern definition. Notably, localized fusion of residual silver at the edges is observed, effectively sealing the ends of conductive yarns and suppressing fraying. Cross-sectional images confirm complete removal of the metallic layer with minimal thermal deformation of the polyester substrate, demonstrating highly selective processing. At high-energy density (30 J·cm^−2^), the laser–material interaction transitions from selective ablation to substrate degradation. Top-view images reveal extensive darkening, fiber charring, and fusion of both silver and polyester fibers. While the conductive layer is fully removed, the underlying textile structure exhibits significant thermal damage. Cross-sectional imaging shows partial collapse of the woven architecture and irreversible deformation of the polymer fibers, indicating that the applied energy exceeds the thermal tolerance of the material. Overall, these results demonstrate that laser energy densities within the optimal regime (10 J·cm^−2^) enable precise and selective removal of conductive silver fibers while preserving the mechanical integrity and structural functionality of the textile substrate. This processing window is critical for achieving high-resolution circuit patterning in MFs and forms the basis for subsequent device fabrication.

To quantify the effect of laser processing on electrical performance, the sheet resistance of laser-ablated metallized fabric (LA-MF) was systematically measured using a four-point probe technique across a range of laser energy densities, applied along both warp and weft directions. Measurements were initially performed along the warp direction with laser scanning aligned in the same orientation to evaluate directional effects within the patterned textile architecture (Fig. 2d and Fig. S1a). The pristine MF exhibited a low sheet resistance of approximately 2 mΩ/sq., consistent with continuous conductive pathways formed by the interwoven silver yarns. A clear and monotonic increase in sheet resistance was observed with increasing laser energy density, reflecting progressive disruption of the conductive network. In the low-energy regime (0–1.75 J·cm^−2^, blue region), the measured sheet resistance remained relatively low, ranging from approximately 3.7 to 100 Ω/sq. Despite partial increases, these values indicate that continuous electrical pathways were largely preserved due to insufficient ablation of the silver fibers, resulting in residual conductivity across the processed regions. In the intermediate regime (1.8–7.5 J·cm^−2^, yellow region), a pronounced and approximately linear increase in sheet resistance was observed with increasing energy density. The resistance increased from approximately 0.1–95 kΩ/sq., corresponding to progressive fragmentation and partial removal of the conductive fibers. This regime reflects a transition from continuous conduction to disrupted, percolative pathways, where residual metallic connections still enable limited charge transport. Upon entering the optimal processing window (8–26.5 J·cm^−2^, green region), the sheet resistance exceeded 1 MΩ/sq., effectively reaching the measurement limit of the system and indicating electrical insulation. This behavior confirms complete removal of the conductive silver layer within the ablated regions, while preserving the underlying nonconductive polyester substrate. The transition to insulating behavior is consistent with the morphological observations of clean and uniform ablation discussed previously.

At higher energy densities (27–40 J·cm^−2^, red region), the samples continued to exhibit insulating behavior despite evidence of thermal degradation and partial carbonization of the underlying polymer fibers. Notably, no measurable conductive pathways were observed in these regions, indicating that carbonized residues did not contribute to electrical conduction under the tested conditions. A similar trend in electrical response was observed for samples processed along the weft direction (Fig. 2d and Fig. S1b), demonstrating that the evolution of electrical sheet resistance with laser energy density is largely independent of processing direction. The consistent behavior across both orientations indicates that laser ablation effectively disrupts conductive yarn networks regardless of their alignment within the woven structure. This behavior is also influenced by the woven architecture and weaving density of the MF, which affect the spacing and interconnectivity of the conductive silver yarn network. Collectively, these results establish a well-defined operational window between 8 and 26.5 J·cm^−2^, within which selective laser ablation enables complete electrical isolation of patterned regions while preserving the structural integrity of the textile substrate. Additionally, localized thermal effects at the ablation boundaries promote partial fusion of residual silver at fiber ends, which may further enhance mechanical stability by reducing fraying at the edges of patterned conductive traces.

### Reproducibility, Process Robustness, and Patterning Resolution

To comprehensively evaluate the manufacturability of the laser-patterning approach, the reproducibility, robustness to geometric variations, and achievable patterning resolution were systematically investigated. These studies collectively assess the reliability of the process under realistic fabrication conditions and define the practical limits for circuit patterning on MFs.

#### Process Reproducibility

To assess reproducibility under identical processing conditions, a design-of-experiments (DOE) study was performed using four independent MF samples subjected to the same laser power and scanning speed parameters. A 10 × 10 checkerboard pattern consisting of 1 cm^2^ (100 features per sample) was patterned onto each fabric specimen, as shown in Fig. S2a–d. Visual inspection of the processed samples revealed highly consistent ablation quality across all specimens, with no discernible variation in feature geometry, edge definition, or pattern fidelity. To quantitatively evaluate reproducibility, electrical sheet resistance measurements were obtained for all 100 patterned regions on each sample. The resulting datasets were statistically analyzed using one-way ANOVA based on the F-statistic to compare inter-sample variation relative to intra-sample variation. As shown in Fig. S2e, the calculated F-statistics were consistently below the critical threshold value of 10.13 (*p* > 0.05), indicating no statistically significant differences between samples. Specifically, the median F value was approximately 0.34, and the maximum observed finite F value was 3.57, both well below the significance threshold. These results demonstrate that the laser-patterning process exhibits high reproducibility, with minimal variability in electrical performance across independently processed samples.

#### Out-of-Plane Processing Robustness

To evaluate the robustness of the laser-ablation process under non-planar surface conditions representative of potential variations encountered in large-scale or roll-to-roll manufacturing, MF samples were processed under controlled out-of-plane geometries. In this study, the fabric was mounted in a slanted configuration such that the surface height varied continuously along the laser scanning direction, as illustrated in Fig. S3a. Five inclination angles (0.5°, 1.0°, 1.5°, 2.0°, and 2.5°) were investigated using the previously identified optimal laser energy density (10 J·cm^−2^). This configuration enabled systematic evaluation of the effect of focal plane deviation and height variation on ablation performance.

Optical imaging (Fig. S3b–f) revealed a progressive dependence of ablation uniformity on inclination angle. At lower inclinations (0.5° and 1.0°), the ablated regions remained largely uniform across the majority of the processed area, with only minor degradation observed at the far end of the sample where the surface deviated most from the focal plane. These results indicate that the laser maintains effective energy delivery within a limited out-of-plane tolerance. As the inclination increased to 1.5° and 2.0°, gradual variations in surface morphology became evident along the scanning direction, reflecting increasing deviation from the optimal focal position. At the highest inclination (2.5°), pronounced non-uniformity in ablation was observed, corresponding to significant defocusing and reduced energy density at the sample surface. To quantitatively assess the effect of focal height variation, sheet resistance was measured at multiple positions along the inclined samples. As shown in Fig. S3g, three distinct regimes of ablation behavior were identified as a function of out-of-plane displacement. In the first regime (0–0.6 mm defocus, green region), complete ablation of the conductive layer was achieved, with sheet resistance exceeding 1 MΩ/sq., indicating effective electrical insulation. This regime defines the acceptable tolerance for surface height variation during processing, within which consistent and reliable metal removal is maintained. In the second regime (0.6–1.5 mm defocus, blue region), partial ablation was observed, characterized by intermediate sheet resistance values. This behavior is attributed to a reduction in local energy density due to beam defocusing and an increase in laser spot size, resulting in incomplete removal of the conductive fibers. In the third regime (> 1.5 mm defocus), minimal ablation occurred, and the measured sheet resistance approached approximately 25 Ω/sq., indicating retention of conductive pathways and insufficient energy delivery to induce material removal. Collectively, these results demonstrate that the laser-patterning process exhibits a well-defined tolerance to surface height variation, with reliable and complete ablation achieved within a defocus range of approximately 0–0.6 mm. This level of robustness is particularly important for scalable textile processing, where perfect planarity cannot be guaranteed.

#### Patterning Resolution

To determine the achievable patterning resolution and dimensional fidelity under optimal laser processing conditions (10 J·cm^−2^), a series of test structures with progressively reduced feature sizes were fabricated on the MF. This study enabled evaluation of the minimum trace width and spacing that could be reliably patterned while preserving textile integrity and electrical functionality. Given a laser spot size of approximately 30 µm, selective removal of individual silver yarns (140–150 µm in diameter) was achieved through multiple raster passes (typically 4–5 passes). The achievable resolution is influenced by the woven architecture of the MF, including yarn spacing and interlacing structure, which impose practical limits on feature definition and continuity. Due to the absence of real-time optical alignment and imaging in the laser system, precise positioning of ultra-fine features is limited. Under these conditions, conductive traces with widths of (400 ± 20) µm were reproducibly fabricated without inducing damage to adjacent fibers or disrupting electrical continuity. At this scale, individual yarn pathways can be selectively defined while maintaining mechanical anchoring through localized fusion of adjacent fibers.

Microscopic analysis (Fig. S4) confirmed sharp edge definition and spatially confined removal of silver filaments across patterned features ranging from (400 ± 20) to (1200 ± 50) µm in width. These results demonstrate high pattern fidelity across a range of practically relevant dimensions. Patterning resolution was further evaluated for both warp- and weft-oriented processing (Fig. S5a,b). Conductive traces with widths ranging from 0.4 to 1.0 mm, representative of typical requirements for wearable and flexible electronics, were successfully fabricated in both directions. Electrical characterization revealed the expected inverse relationship between sheet resistance and trace width, consistent with reduced current-carrying cross-section in narrower traces. Notably, only 4–7% variation in sheet resistance was observed between corresponding warp- and weft-patterned traces (Fig. S5c). This minimal variation in electrical performance can be attributed to the uniform distribution and comparable density of conductive silver yarns in both warp and weft directions within the woven architecture. As a result, the effective conductive pathways and electrical transport characteristics remain largely isotropic, leading to consistent electrical behavior irrespective of processing orientation.

### Morphological and Elemental Characterization

**Fig. 3:**
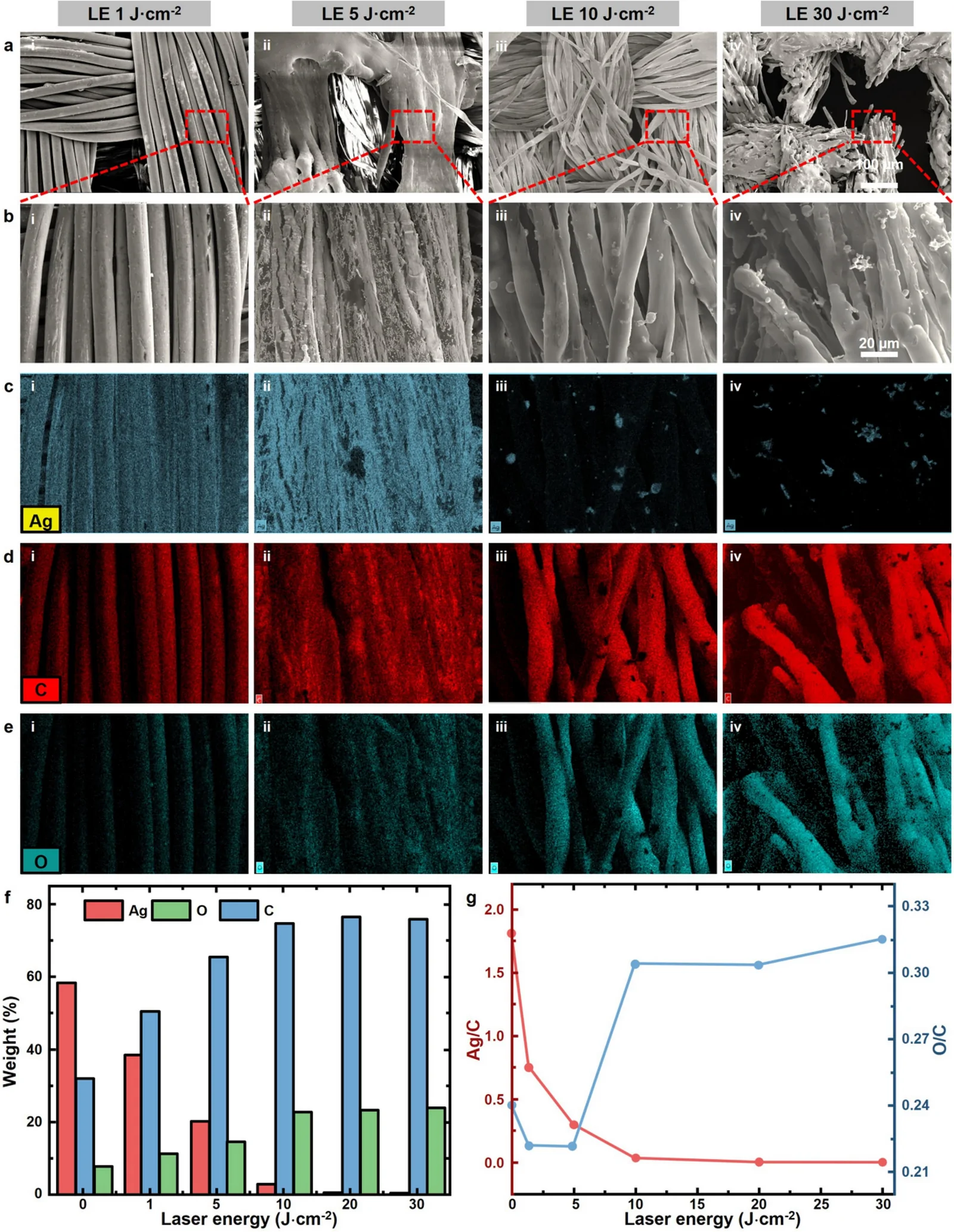
Morphological and elemental characterization of MF processed at different laser energy densities. **a** Low-magnification and **b** high-magnification SEM images of (i) pristine MF, and samples processed at energy densities corresponding to (ii) partial silver removal, (iii) optimized selective ablation, and (iv) thermally induced substrate degradation. EDS elemental maps showing the spatial distribution of **c** silver (Ag), **d** carbon (C), and **e** oxygen (O). **f** Quantitative elemental composition (wt.%) of Ag, C, and O as a function of laser energy density. **g** Variation in the Ag/C and O/C ratios, highlighting progressive removal of the conductive silver layer and increasing exposure and oxidation of the underlying polyester substrate

The surface morphology and compositional evolution of pristine and LA-MF were systematically investigated using scanning electron microscopy (SEM) and energy-dispersive spectroscopy (EDS). These analyses were conducted to elucidate the effects of laser energy density on structural modification and elemental distribution within the MF. SEM micrographs of representative samples processed under four distinct laser energy densities (i) 1 J·cm^−2^ (low), (ii) 5 J·cm^−2^ (intermediate), (iii) 10 J·cm^−2^ (optimal), and (iv) 30 J·cm^−2^ (high) are presented in Fig. 3a,b. At low-energy density (1 J·cm^−2^), the silver yarns remained structurally intact, exhibiting smooth filament surfaces, well-defined strand bundling, and continuous metallic coverage along the yarn length (Figs. 3a(i), 3b(i)). No discernible morphological changes or evidence of ablation were observed, confirming that the applied energy was below the threshold required for material removal. At intermediate energy density (5 J·cm^−2^), partial disruption of the silver filaments was observed. SEM images revealed localized melting, filament deformation, and the formation of irregular agglomerates of resolidified silver redeposited onto the underlying polyester fibers (Fig. 3a(ii)). High-magnification imaging (Fig. 3b(ii)) showed roughened surface morphology and filament coalescence, indicating melt-driven redistribution rather than complete ablation. These features are consistent with insufficient energy input to fully vaporize the metallic layer. In contrast, samples processed at the optimal energy density (10 J·cm^−2^) exhibited clean and uniform removal of the silver yarns. SEM analysis confirmed complete ablation of the conductive layer, exposing the underlying polyester fibers while preserving their structural integrity (Figs. 3a(iii), 3b(iii)). The exposed fibers retained their original morphology, with minimal surface roughening and no evidence of collapse or thermal degradation. These observations demonstrate highly selective material removal under optimized processing conditions.

At high-energy density (30 J·cm^−2^), the laser–material interaction transitioned from selective ablation to substrate degradation. SEM images revealed extensive thermal damage, including fiber fusion, structural collapse, and loss of the characteristic fibrous architecture (Figs. 3a(iv), 3b(iv)). The resulting morphology appeared as a fused and brittle network with significant deformation, indicating that the applied energy exceeded the thermal tolerance of the textile substrate. To further investigate compositional changes, EDS elemental mapping was performed to analyze the spatial distribution of silver (Ag), carbon (C), and oxygen (O) across the processed samples (Fig. 3c–e). At low-energy density (1 J·cm^−2^), strong and uniform Ag signals were observed (Fig. 3c(i)), consistent with intact silver yarns. The carbon signal (Fig. 3d(i)**)** corresponded to the underlying polyester substrate, while oxygen levels remained minimal (Fig. 3e(i)), indicating negligible oxidation or compositional change. At intermediate energy density (5 J·cm^−2^), a moderate reduction in Ag signal intensity was observed (Fig. 3c(ii)), accompanied by increased carbon and oxygen signals (Figs. 3d(ii), 3e(ii)). This trend is attributed to partial removal of silver and increased exposure of the polymer substrate, along with localized oxidation induced by laser heating in ambient conditions. In the optimal regime (10 J·cm^−2^), EDS mapping revealed near-complete removal of silver, with only trace residual particles (< 5 µm) observed on the polyester fibers (Fig. 3c(iii)). These residuals are attributed to minor redeposition of vaporized silver during ablation. In contrast, carbon and oxygen signals became dominant (Figs. 3d(iii), 3e(iii)), reflecting full exposure of the underlying polyester matrix. The uniformity of these signals indicates that the substrate remained structurally intact without significant chemical degradation. At the highest energy density (30 J·cm^−2^), Ag signals remained negligible, while carbon and oxygen signals increased significantly (Figs. 3c(iv), 3d(iv), 3e(iv)). This behavior is consistent with thermal degradation and partial carbonization of the polyester fibers, resulting from excessive laser energy input and prolonged localized heating. Quantitative EDS analysis (Fig. 3f) further confirmed these trends. The atomic percentage of Ag decreased progressively from approximately 58% at low-energy densities to 1% at the highest energy conditions. Correspondingly, carbon content increased from approximately 32 to 76%, while oxygen content increased from approximately 7–20%, reflecting progressive removal of the metallic layer and exposure of the polymer substrate.

To better interpret these compositional changes, elemental ratios were evaluated as a function of laser energy density (Fig. 3g). The Ag-to-C ratio decreased significantly from approximately 1.80 in pristine samples to 0.75 and 0.03 at intermediate and optimal energy densities (1 and 10 J·cm^−2^, respectively), and further to approximately 0.03 at higher energies (20–30 J·cm^−2^). This trend confirms effective removal of the conductive silver layer and increasing dominance of the underlying carbon-rich textile. Simultaneously, the O-to-C ratio increased from 0.24 in pristine samples to approximately 0.32 at the highest energy density, indicating progressive oxidation of the exposed substrate. This increase is attributed to enhanced surface reactivity and localized heating in the presence of ambient oxygen during laser processing. Collectively, these morphological and compositional analyses confirm that laser energy density plays a critical role in governing the transition from intact conductive networks to selective ablation and ultimately to substrate degradation. The optimal processing regime (10 J·cm^−2^) enables complete removal of the conductive layer while preserving the structural and chemical integrity of the textile, thereby supporting the development of high-performance, laser-patterned e-textiles.

### Mechanical and Breathability Characterization

To evaluate the effect of laser ablation on the mechanical integrity and structural robustness of the MF, uniaxial tensile testing was performed on dog-bone-shaped specimens processed under a range of laser energy densities. In all samples, laser patterning was confined to the gauge section in order to isolate the influence of the ablated region on the measured mechanical response. Mechanical behavior was evaluated for specimens patterned along both the warp and weft directions of the woven architecture.

**Fig. 4:**
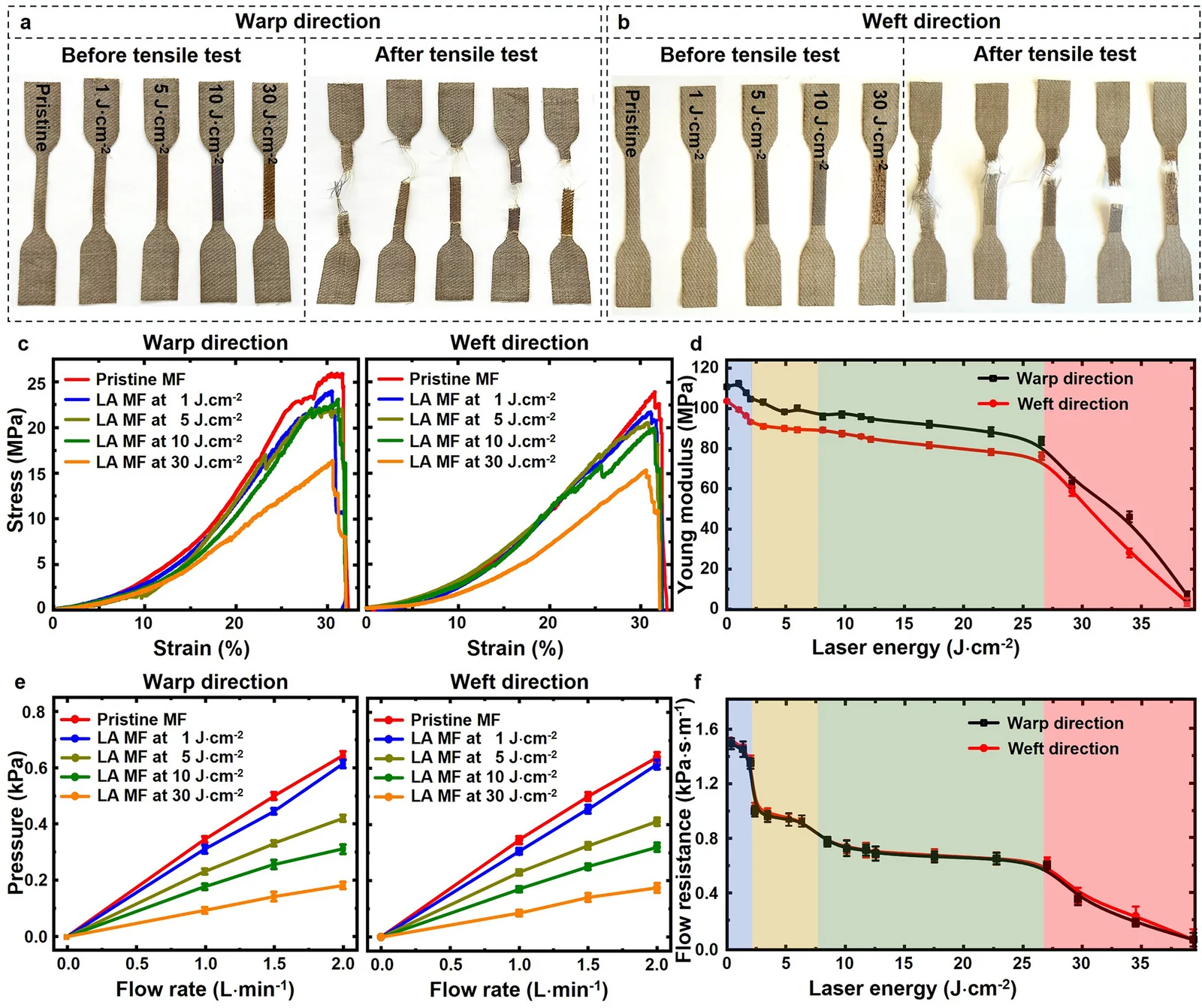
Mechanical and breathability characterization of LA-MF processed along the warp and weft directions. **a,b** Representative images of pristine MF and samples laser-ablated at energy densities of 1, 5, 10, and 30 J·cm^−2^ before and after tensile testing, illustrating the corresponding fracture morphology in the warp and weft orientations. **c** Representative stress–strain curves of specimens processed under different laser conditions. **d** Young’s modulus as a function of laser energy density for warp- and weft-oriented samples. **e** Pressure drop as a function of airflow rate for pristine and laser-ablated MF, used to determine airflow resistance and breathability. **f** Extracted airflow resistance of MF as a function of laser energy density for both processing orientations. Data are presented as mean values from five independent samples (*n* = 5)

Figure 4a,b presents representative images of pristine MF and LA-MF processed at 1, 5, 10, and 30 J·cm^−2^ before and after tensile testing in both warp- and weft-oriented configurations. The fracture morphology provides important insight into the underlying deformation and failure mechanisms of the hybrid textile-metal architecture. In pristine MF specimens, the fracture zone exhibits extensive yarn decrimping, fiber reorientation, and progressive axial elongation prior to failure. At the point of rupture, both the polyester yarns and conductive silver filaments display characteristic frayed filament bundles, fiber pullout, and brush-like fracture ends, indicating a ductile and energy-dissipative failure process. The silver filaments exhibit localized plastic deformation and tensile rupture, while the polyester fibers undergo fibrillation and progressive tensile fracture. Together, these observations confirm that both the polymeric and metallic yarn networks actively contribute to the load-bearing capability of the MF.

Samples processed at a low laser energy density of 1 J·cm^−2^ exhibited fracture morphologies nearly identical to those of the pristine material. Pronounced fraying, brush-like fiber ends, and progressive filament pullout were observed at the fracture surfaces, indicating that the applied laser energy was below the threshold required to significantly alter either the conductive silver yarns or the underlying polyester structure. These results demonstrate that sub-threshold laser exposure has negligible impact on the mechanical performance of the fabric.

At an intermediate energy density of 5 J·cm^−2^, the fracture morphology changed noticeably. The broken ends of the specimens were dominated by polyester fibrillation, while the characteristic frayed metallic filament bundles were largely absent. This behavior is consistent with partial melting and discontinuity of the silver filaments, which no longer act as continuous load-bearing elements. Residual silver, although still present, appeared as localized molten deposits adhered to the polyester fibers and contributed minimally to tensile reinforcement. A similar fracture morphology was observed in samples processed at the optimized energy density of 10 J·cm^−2^, where complete removal of the conductive silver layer resulted in fracture surfaces composed almost exclusively of polymer fibers. Importantly, the polyester yarns retained extensive fibrillation and brush-like fracture ends, indicating that the underlying textile structure remained mechanically intact and continued to deform in a ductile manner despite complete ablation of the conductive layer.

In contrast, specimens processed at a high laser energy density of 30 J·cm^−2^ exhibited a markedly different and more brittle failure mode. The fracture surfaces showed minimal fiber pullout, limited fibrillation, and an absence of the characteristic brush-like morphology observed in lower-energy samples. Instead, the fibers appeared fused, embrittled, and sharply fractured, with little evidence of progressive elongation prior to rupture. These features indicate severe thermal degradation of the polyester network, resulting in loss of fiber integrity and a substantial reduction in the fabric’s ability to dissipate mechanical energy.

To quantitatively assess the effect of laser processing on tensile performance, stress–strain curves were obtained for samples processed across the full range of laser energy densities in both warp and weft directions. Representative stress–strain curves for four characteristic processing conditions are shown in Fig. 4c. In all cases, the initial low-stiffness region corresponds to crimp removal (decrimping) and progressive alignment of the woven yarns, representing a compliance-dominated response before effective load transfer occurs within the fiber network.

Following this toe region, pristine MF and samples processed at 1 J·cm^−2^ exhibited steep elastic slopes and high ultimate tensile strengths, reaching approximately 25 MPa in the warp direction and 24.5 MPa in the weft direction. These values reflect the combined reinforcement provided by both the polyester substrate and the conductive silver yarn network. As the laser energy density increased to 5 and 10 J·cm^−2^, a modest reduction in tensile strength and stiffness was observed, corresponding to progressive disruption and eventual removal of the silver fibers. Specifically, the ultimate tensile strength decreased by approximately 1.8 MPa in the warp direction and 1.1 MPa in the weft direction relative to the pristine material. Despite this reduction, the samples maintained a highly ductile stress–strain response, confirming that the polyester fibers remained the dominant structural component and preserved the overall mechanical behavior of the textile.

At the highest energy density (30 J·cm^−2^), the stress–strain response exhibited a pronounced reduction in both elastic slope and ultimate tensile strength, with decreases of approximately 15 MPa in the warp direction and 14.8 MPa in the weft direction. This substantial loss in mechanical performance confirms that excessive laser energy induces significant thermal damage to the polyester fiber network, compromising the principal load-bearing structure of the fabric.

To further quantify the influence of laser processing on material stiffness, Young’s modulus was determined from the linear elastic portion of the stress–strain curves, excluding the initial toe region associated with yarn decrimping. The resulting values are summarized in Fig. 4d. Both warp- and weft-oriented specimens exhibited an overall decrease in modulus with increasing laser energy density; however, distinct plateau regions were observed corresponding to the previously identified laser-processing regimes.

Along the warp direction, pristine MF exhibited a Young’s modulus of approximately 110 MPa. Within the low-energy regime (0–1.75 J·cm^−2^), the modulus decreased modestly to approximately 102 MPa, indicating minimal structural modification. In the intermediate regime (1.8–7.5 J·cm^−2^), the modulus remained relatively constant at approximately 96 MPa, suggesting that partially melted and discontinuous silver residues no longer contributed significantly to the tensile response. Within the optimized selective-ablation regime (8–26.5 J·cm^−2^), the modulus remained remarkably stable, decreasing by less than 12 MPa across the entire processing window. This result demonstrates that complete removal of the conductive silver layer can be achieved while preserving the structural integrity and stiffness of the underlying polyester network. In contrast, within the high-energy regime (27–40 J·cm^−2^), the Young’s modulus decreased sharply from 64 MPa to as low as 6 MPa, reflecting severe thermal degradation and embrittlement of the polyester fibers.

A nearly identical trend was observed in the weft direction, with modulus values differing by only 3 to 5% relative to the corresponding warp-oriented specimens. Furthermore, within the optimized processing window (8–26.5 J·cm^−2^), the ultimate tensile strength of samples processed in both orientations varied by only approximately 5–10%. This high degree of consistency confirms that the laser-patterning process preserves the mechanical integrity of the MF irrespective of processing direction.

Collectively, these results demonstrate that selective laser ablation within the optimized energy–density range enables complete removal of the conductive silver fibers while maintaining the intrinsic tensile behavior, ductility, and stiffness of the underlying textile substrate. The preservation of mechanical properties within this processing window can be attributed to the highly selective absorption and rapid vaporization of the silver fibers at the Nd:YAG laser wavelength (1.06 µm), which minimizes heat transfer to the polyester fibers and thereby limits thermal damage to the load-bearing textile architecture.

To evaluate the effect of laser ablation on the functional breathability of the MF, airflow resistance was measured for pristine and LA-MF samples processed along both the warp and weft directions in accordance with ASTM D737. During testing, a controlled volumetric airflow ranging from 0 to 2 L·min^−1^ was applied normal to the fabric surface, and the resulting pressure differential across the sample was recorded. Airflow resistance was determined from the slope of the pressure drop versus volumetric flow rate relationship, normalized by the exposed sample area, thereby providing a quantitative measure of the resistance of the textile to air transport.

As shown in Fig. 4e, all samples exhibited an approximately linear increase in pressure differential with increasing airflow rate, consistent with Darcy-type flow through porous fibrous media. For both warp- and weft-oriented specimens, the pristine MF displayed the steepest slope, corresponding to the highest airflow resistance. This behavior is attributed to the presence of continuous silver yarns interwoven within the polyester substrate, which increase the effective packing density of the textile and partially obstruct inter-fiber voids, thereby restricting airflow through the fabric.

Samples processed at low laser energy density (1 J·cm^−2^) exhibited only a modest reduction in airflow resistance, with values approximately 15–21% lower than those of the pristine MF. This limited change is consistent with the morphological observations showing minimal disruption of the conductive yarn network under sub-threshold laser conditions. As the laser energy density increased to 5 and 10 J·cm^−2^, the airflow resistance decreased substantially to approximately 50–55% of the pristine value. Notably, the resistance values at 5 and 10 J·cm^−2^ were relatively similar, indicating that partial melting and disruption of the silver yarns at 5 J·cm^−2^ and complete removal at 10 J·cm^−2^ both significantly increase the openness of the textile structure and reduce obstruction to air transport.

In contrast, samples processed at 30 J·cm^−2^ exhibited the lowest airflow resistance, corresponding to a reduction greater than 80% relative to the pristine MF in both processing directions. This dramatic decrease is attributed to excessive thermal degradation of the polyester substrate, which disrupts the woven architecture and produces a highly porous, structurally damaged network with minimal resistance to airflow.

A summary of the extracted airflow resistance values as a function of laser energy density is presented in Fig. 4f. The trends obtained for warp- and weft-oriented processing were nearly identical, with less than (± 1)% variation across the entire range of laser conditions, indicating that the effect of laser processing on permeability is essentially independent of scanning direction. This high degree of overlap is consistent with the similar distribution and packing density of conductive silver yarns in both orientations of the woven structure.

Several distinct transitions in airflow resistance were observed that closely correspond to the previously identified laser-processing regimes. The pristine MF exhibited an airflow resistance of approximately 1.5 kPa·s·m^−1^, and this value remained relatively unchanged throughout the low-energy regime (0–1.75 J·cm^−2^), where no significant structural modification occurred. Upon entering the intermediate regime (1.8–7.5 J·cm^−2^), the airflow resistance decreased to approximately 30% to 40% of the pristine value. This reduction is attributed to partial melting and fragmentation of the silver yarns, which disrupts the dense conductive network and increases the effective porosity of the textile, even though residual metallic material remains attached to the polyester fibers.

Within the optimized selective-ablation regime (8–26.5 J·cm^−2^), a further stepwise reduction of approximately 10% in airflow resistance was observed. This additional decrease corresponds to complete removal of the conductive silver fibers, resulting in exposure of the underlying polyester network and elimination of the metallic contribution to flow restriction. Importantly, despite this substantial increase in breathability, the tensile properties of the textile remained largely unchanged, demonstrating that selective removal of the conductive layer does not compromise the structural integrity of the load-bearing polyester fibers.

At higher laser energy densities (> 27 J·cm^−2^), airflow resistance decreased rapidly with increasing energy input. This behavior is attributed to thermal degradation, fusion, and partial vaporization of the polyester fibers, which disrupt the woven architecture and create large voids within the textile. Collectively, these results demonstrate that selective laser ablation not only enables precise patterning of conductive pathways but also significantly enhances the breathability of the MF by removing dense metallic yarns while preserving the intrinsic porous structure of the textile within the optimized processing window.

### Wash Durability and Reliability Characterization

**Fig. 5:**
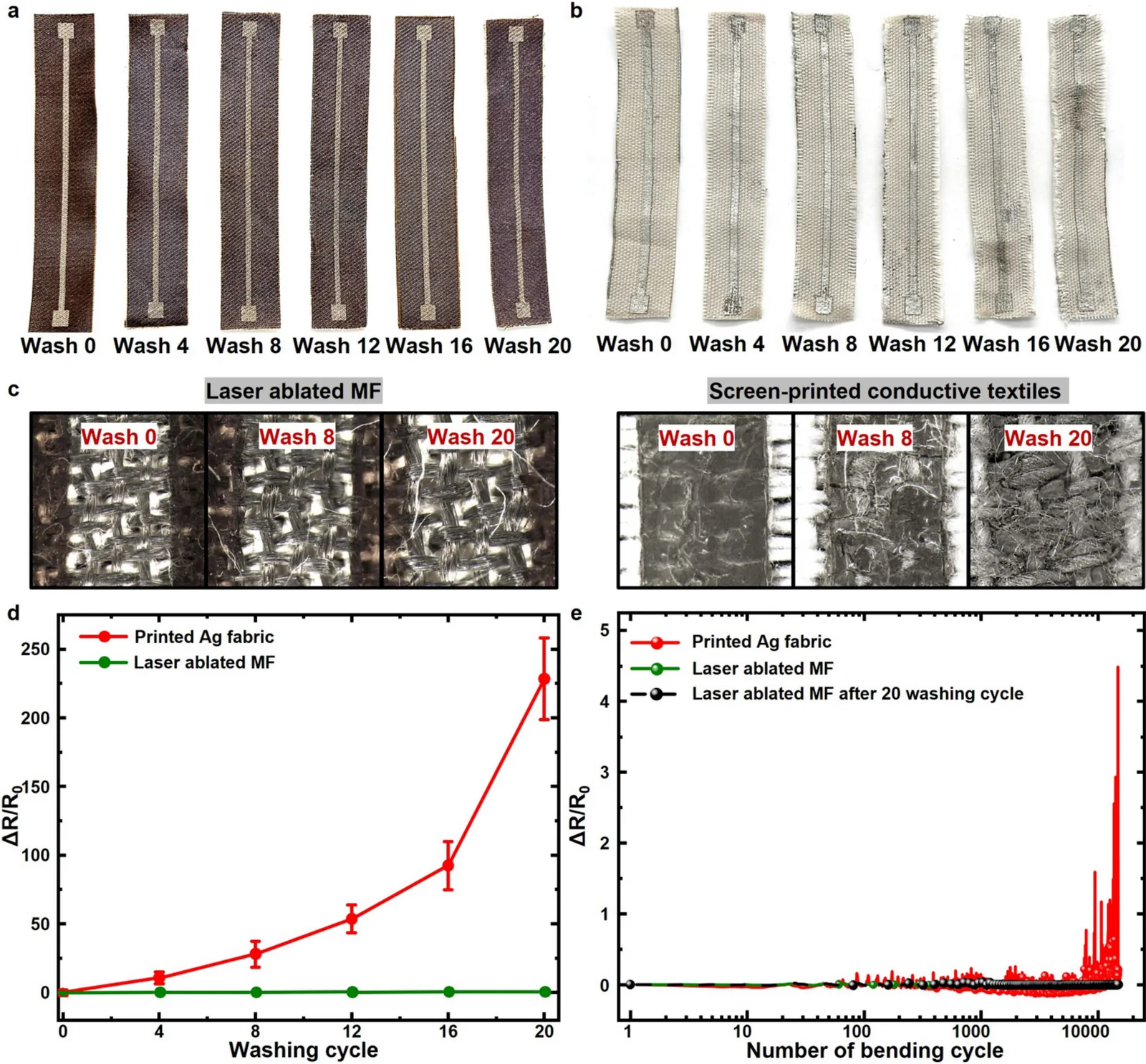
Wash durability and mechanical reliability of LA-MF compared with screen-printed silver traces. **a** LA-MF samples and **b** screen-printed silver traces before and after repeated washing cycles. **c** Optical microscopy of conductive regions before and after laundering, showing preservation of the woven silver network in LA-MF and progressive cracking, delamination, and material loss in screen-printed traces. **d** Normalized resistance change (Δ*R/R₀*) as a function of washing cycles, demonstrating minimal variation in LA-MF conductivity compared with the substantial resistance increase observed in screen-printed samples. **e** Normalized resistance changes during cyclic bending for LA-MF before and after 20 washing cycles, compared with freshly prepared screen-printed traces, highlighting the superior mechanical durability and electrical stability of the laser-patterned textile conductors

To evaluate the laundering durability of the LA-MF, changes in electrical performance and structural integrity were systematically monitored following repeated washing under standardized conditions. Straight conductive traces were patterned on MF using the optimized laser energy density of 10 J·cm^−2^ and subjected to sequential laundering cycles. After each cycle, the electrical resistance was measured to quantify retention of conductive performance (Fig. 5a). For comparison, conductive traces of identical geometry were fabricated on woven polyester fabric using conventional screen printing with a commercial silver paste and exposed to the same washing protocol (Fig. 5b).

The LA-MF samples exhibited no visually detectable changes in trace geometry, edge definition, or electrical continuity after repeated laundering. Even after 20 wash cycles, the conductive pathways remained uniform and sharply defined, indicating excellent structural and functional robustness. Optical microscopy further confirmed that the conductive silver yarns remained mechanically interlocked within the woven textile architecture, with no evidence of cracking, peeling, delamination, or fraying either within the conductive traces or at the boundaries between laser-patterned and non-patterned regions (Fig. 5c). Because the conductive pathways are formed by continuous, multifilament silver yarns intrinsically integrated into the fabric, the conductive network remains mechanically anchored to the underlying polyester substrate and is not susceptible to the interfacial failure mechanisms commonly associated with surface-deposited coatings.

Consistent with these observations, the electrical resistance of the LA-MF samples remained highly stable throughout the laundering sequence, varying by less than approximately 5% relative to the initial value after 20 washing cycles (Fig. 5d). This minimal change demonstrates excellent retention of electrical conductivity under repeated exposure to water, detergent, and mechanical agitation. In contrast, the screen-printed control samples exhibited progressive structural degradation and substantial loss of electrical performance with increasing wash cycles. Optical microscopy revealed extensive cracking of the printed silver layer, interfacial delamination from the polyester fibers, and progressive material loss from the fabric surface, resulting in discontinuities within the conductive traces and exposure of the underlying fibers (Fig. 5c). This degradation is attributed to the particulate nature of the printed conductive layer and the dependence on polymeric binders to maintain adhesion. Repeated laundering introduces moisture, detergent exposure, abrasion, and cyclic deformation, which promote binder swelling, interfacial debonding, crack formation, and eventual loss of conductive pathways. Consistent with these structural changes, the electrical resistance of the screen-printed samples increased by approximately 50-fold after eight washing cycles and exceeded 250 times the initial resistance after 20 cycles (Fig. 5d), indicating severe deterioration of the printed conductive network. These results clearly demonstrate the superior wash durability, structural integrity, and electrical stability of LA-MF relative to conventional screen-printed textile conductors.

To further assess mechanical reliability, cyclic bending tests were performed on LA-MF samples fabricated at the optimized laser energy density of 10 J·cm^−2^, both before washing and after 20 laundering cycles. Their performance was compared with freshly prepared screen-printed traces of identical dimensions. Electrical resistance was continuously monitored during 10000 bending cycles to evaluate the ability of each conductive architecture to maintain electrical continuity under repeated deformation.

As shown in Fig. 5e, the electrical resistance of the screen-printed samples remained relatively constant during the initial stages of testing, up to approximately 100 bending cycles. Beyond this point, a progressive increase in resistance was observed, reaching approximately 50% above the initial value after 5000 cycles and increasing to approximately 400% after 10000 cycles. This behavior is indicative of fatigue-induced microcrack formation, crack propagation, and fragmentation within the brittle particulate conductive layer, resulting in progressive disruption of the percolative network.

In contrast, the LA-MF samples exhibited exceptional mechanical durability. The normalized resistance change (Δ*R/R*_*0*_) remained nearly constant throughout the entire 10000-cycle bending test. After 5000 cycles, the resistance increased by only 5%, and after 10000 cycles, the total increase was approximately 6%. Importantly, nearly identical behavior was observed for samples tested after 20 washing cycles, demonstrating that laundering did not compromise their bending reliability.

The outstanding mechanical stability of LA-MF arises from several structural advantages. First, electrical conduction occurs through continuous, multi-stranded silver filaments whose ductile metallic nature and bundled architecture enable effective redistribution of strain and high resistance to flexural fatigue. Second, because the conductive elements are woven directly into the textile matrix, the conductive pathways are mechanically anchored within the fabric rather than merely attached to the surface. Third, the absence of polymeric binders eliminates aging, swelling, and moisture-induced degradation that commonly affect printed conductive coatings. Collectively, these results demonstrate that laser-patterned MFs provide exceptional resistance to both laundering and repeated mechanical deformation. By preserving the native woven conductive architecture and avoiding the interfacial weaknesses inherent to printed conductors, the LA-MF platform delivers durable and stable electrical performance suitable for long-term wearable electronic applications.

To benchmark the performance of the selective laser patterning of MF conductors for wearable electronics, representative fabrication strategies for e-textile conductors are summarized in Table S1. Existing approaches generally fall into three categories: (i) additive deposition methods, including screen printing, inkjet printing, and transfer printing of conductive inks; (ii) fiber- and fabric-based approaches, such as conductive-thread embroidery and lamination of pre-conductive textiles; and (iii) direct textile patterning methods based on photolithography. Each approach has enabled significant advances in wearable sensing, heating, and wireless communication, but often involves trade-offs among electrical conductivity, spatial resolution, mechanical durability, wash stability, and textile comfort.

Printed e-textiles can achieve conductivities on the order of 10^5^–10^6^ S/m and sheet resistances ranging from 0.1 Ω/sq. to several Ω/sq., with feature sizes typically between 0.2 and 1.0 mm [32, 38, 69]. However, because these systems rely on percolative networks of conductive particles embedded in polymeric binders, they are susceptible to cracking, delamination, and resistance drift during repeated bending and laundering, often requiring encapsulation to maintain performance beyond 10–20 wash cycles [70–72]. Conductive-thread embroidery and conductive-fabric lamination overcome some of these limitations by utilizing inherently conductive textile elements, resulting in excellent flexibility and wash durability [73, 74]. Nevertheless, their spatial resolution is constrained by yarn diameter, stitch geometry, and assembly complexity, making them less suitable for dense, PCB-like circuit architectures [47, 75]. Textile photolithography has demonstrated sub-100 µm patterning with excellent bending and wash stability, but requires multistep metallization and wet-chemistry processes that increase fabrication complexity [49, 50, 76].

In comparison, the laser-assisted patterning approach developed in this work provides a practical balance between high performance, fine resolution, and manufacturing simplicity (Fig. S6). By selectively ablating conductive fibers from a pre-metallized textile, the process forms electrically isolated circuit pathways directly within the fabric while preserving continuous metallic conduction in the remaining woven network. The resulting LA-MF exhibits low resistance (approximately 2 mΩ/sq.), feature sizes of approximately 400 µm, stable electrical performance over more than 10,000 bending cycles, and durability exceeding 20 machine-wash cycles, while maintaining the inherent flexibility and breathability of the textile. The mask-free and digitally programmable nature of the process further enables rapid design iteration and scalable fabrication. Collectively, these results position LA-MF as an attractive intermediate between robust textile-native conductors and high-resolution microfabrication methods, offering a versatile route toward mechanically durable and electrically stable fabric-integrated electronic systems.

To further evaluate the comfort, flexibility, and wearability of the MF before and after laser processing, the drape behavior of pristine MF and LA-MF processed at the optimized laser energy density of 10 J·cm^−2^, representative of the selective-ablation operating window (8–26.5 J·cm^−2^), was assessed. The results demonstrated that selective laser removal of the conductive silver fibers produced only a modest increase in drape coefficient (7%), while the overall projected drape profiles remained highly comparable to those of the pristine MF across all tested fabric orientations (0°, 45°, and 90°), as shown in Fig. S7 and Note 1. These observations indicate that the laser-patterning process preserves the inherent flexibility and conformability of the textile despite localized removal of the conductive layer.

In addition, although no noticeable odor was detected by users following laser processing, a systematic volatile organic compound (VOC) analysis was performed to evaluate the potential generation of trace gaseous byproducts during laser ablation. For this assessment, pristine MF and LA-MF samples processed at 10 J·cm^−2^ were placed inside a sealed sensing chamber equipped with a multi-channel VOC gas sensor array, as shown in Fig. S8a,b. The sensor responses were continuously monitored to quantitatively evaluate volatile emissions associated with both pristine and laser-processed fabrics. As shown in Fig. S8c, d, both MF and LA-MF exhibited extremely low VOC emissions, with measured concentrations for alcohols, NO_x_, ammonia, toluene, ethanol, and methane remaining below the detection limit of the sensors (< 1 ppm) and comparable to baseline ambient-air conditions. Furthermore, washing of the fabrics before or after laser processing did not measurably influence the VOC emission behavior.

To further investigate the possibility of trace VOC accumulation over time, the differential sensor response within the sealed chamber was analyzed over a 1 h monitoring period for pristine MF and LA-MF samples before and after washing, as shown in Fig. S8e. Across all sensing channels, the observed variation remained within 3%, indicating negligible generation of detectable volatile compounds from the fabrics. Statistical analysis further confirmed minimal deviation between the tested conditions (*p* > 0.01), demonstrating that the laser-assisted patterning process does not introduce significant odor-related or volatile-emission concerns under the investigated operating conditions.

### Wearable Electrical Impedance Tomography System

**Fig. 6:**
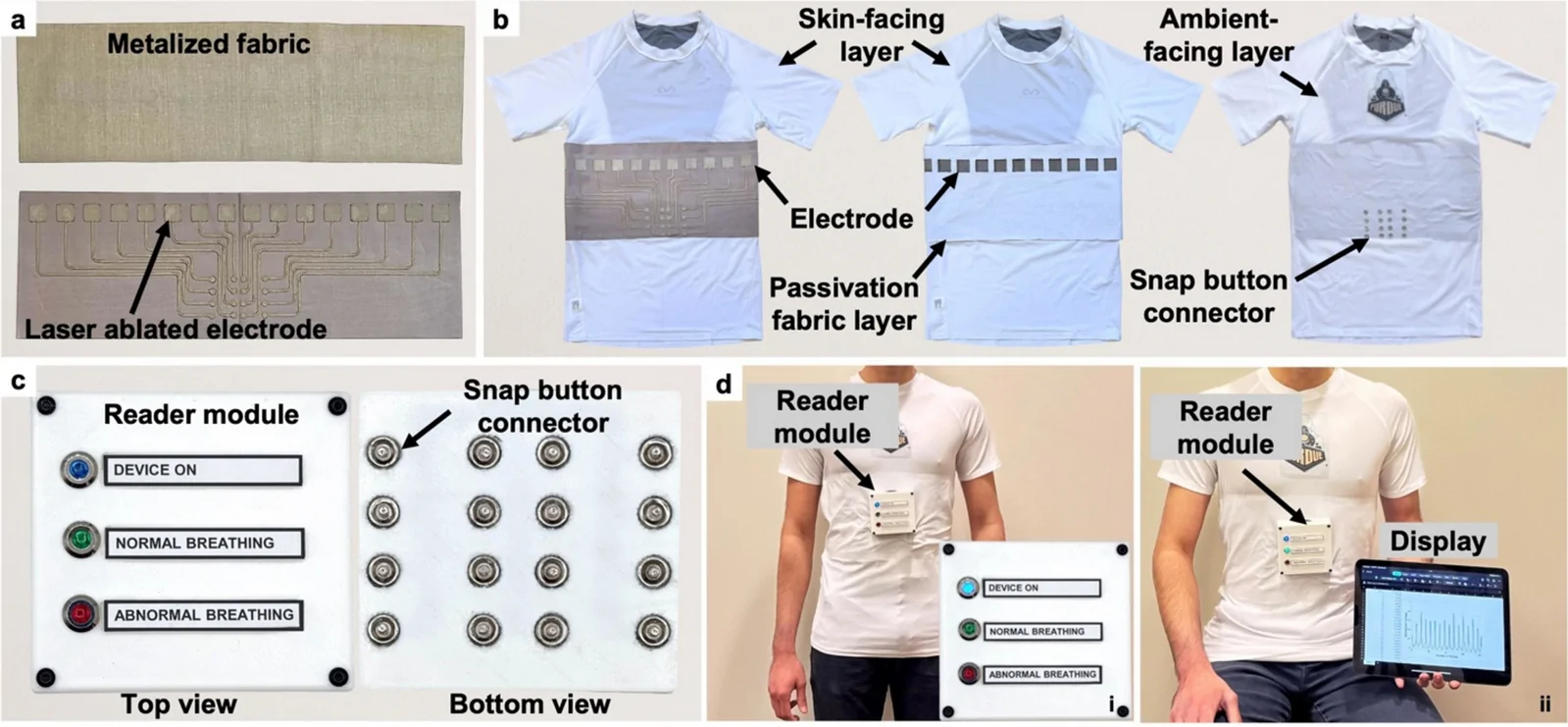
Wearable EIT system integrated into a garment through selective laser patterning of MF. **a** Fabrication of the textile electrode array before and after laser ablation, showing selective removal of conductive silver fibers to define 16 electrodes and their interconnects with high spatial resolution. **b** Multilayer construction of the wearable EIT garment, consisting of a skin-facing electrode layer, a passivation layer with exposed electrode contact regions, and an outer textile layer with integrated snap-button connectors. **c** Portable reader module with status LED and wireless communication, and the underside snap-button array used for modular electrical connection to the garment electrodes. **d** Demonstration of the wearable system during (i) standing and (ii) seated operation, showing real-time acquisition and wireless visualization of respiratory signals

As a proof of concept for the practical implementation of selective laser patterning of MF in large-area wearable electronics, the optimized laser ablation process was used to fabricate a garment-integrated electrode array for electrical impedance tomography (EIT)-based respiratory monitoring. In this application, 16 electrodes and their associated interconnects were directly patterned onto the MF and incorporated into a commercial compression shirt to enable distributed electrical measurements across the thoracoabdominal region for noninvasive assessment of lung activity (Fig. 6a and Fig. S9a,b).

The patterned textile was integrated into a multilayer garment architecture designed to ensure stable skin contact while maintaining electrical insulation between adjacent interconnects. The innermost layer consisted of the laser-patterned electrode array positioned in direct contact with the skin. A second passivation layer composed of breathable cotton fabric was placed above the patterned textile, with laser-defined openings exposing only the electrode contact regions while electrically isolating the conductive interconnects and minimizing the risk of shorting during body motion (Fig. 6b). A third outer textile layer enclosed the assembly while exposing only the snap-button terminals used to interface the garment with a portable electronic acquisition module. This modular connection enabled secure electrical contact and straightforward removal of the electronics during laundering and reuse.

The portable reader module incorporated front-panel status indicators, including a blue light emitting diode (LED) for system power, a green LED indicating normal respiratory activity, and a red LED corresponding to abnormal breathing patterns. The module also included wireless communication capabilities for real-time transmission of measurement data to a tablet or computer for signal visualization and respiratory monitoring (Fig. 6c). Electrical interfacing between the garment and the reader module was achieved through a 16-channel snap-button array aligned with the textile electrode layout. The complete system was comfortably worn under representative conditions, including standing and seated postures, enabling continuous acquisition of physiological signals and wireless transfer of data to an external display (Fig. 6d).

**Fig. 7:**
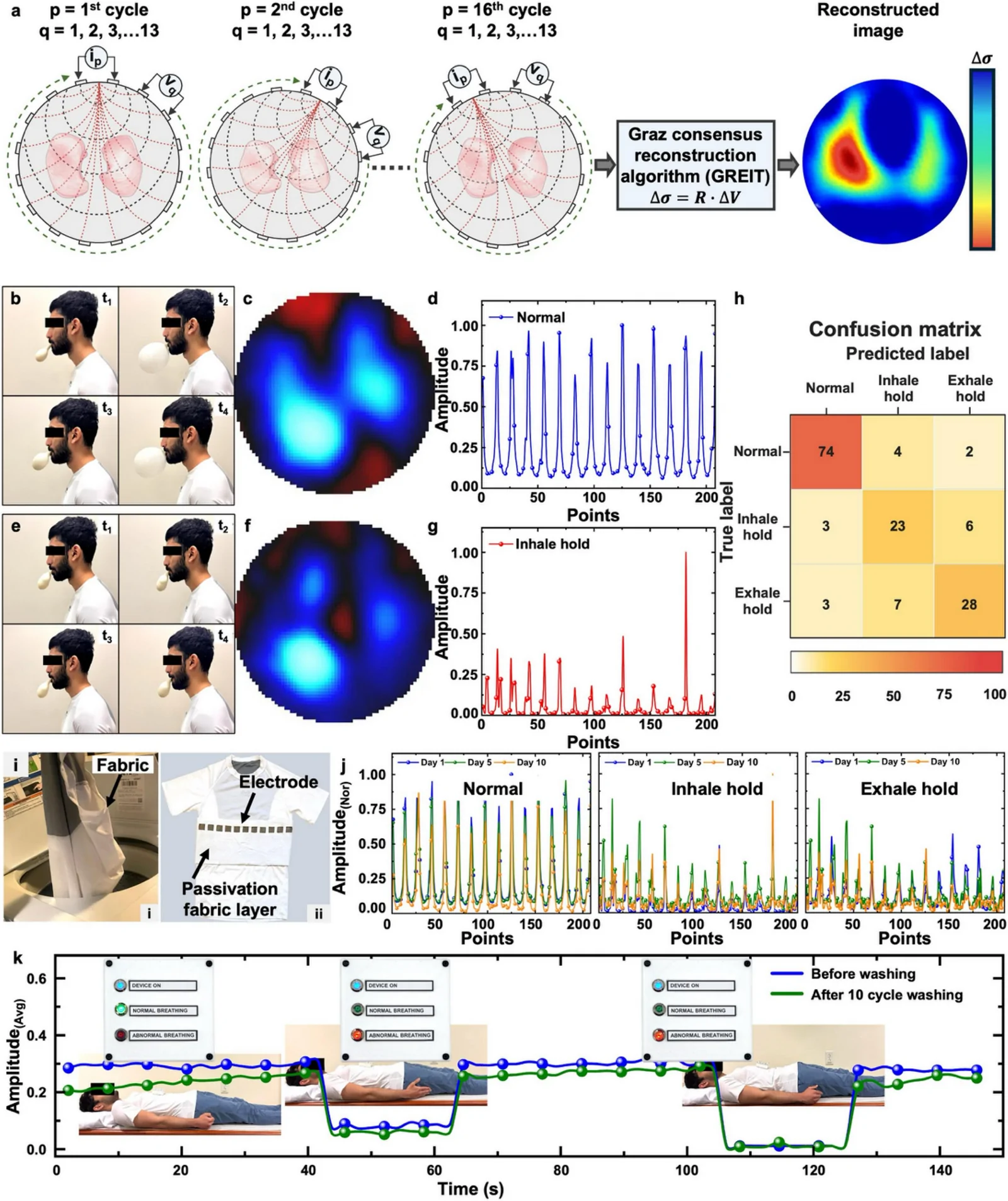
Performance of LA-MF electrodes for wearable EIT-based respiratory monitoring, machine-learning classification, and wash durability. **a** Current-injection and voltage-acquisition sequence across the 16-electrode array used for GREIT-based image reconstruction. **b** Subject wearing the garment during normal breathing. **c** Reconstructed conductivity map under normal breathing. **d** Representative normalized voltage profile during normal respiration. **e** Subject performing an inhale-hold maneuver. **f** Reconstructed conductivity map during inhale-hold. **g** Corresponding normalized voltage profile. **h** Confusion matrix of the neural-network classifier for discrimination of normal breathing, inhale-hold, and exhale-hold. **i** Electrode-integrated garment during machine washing (i) and optical image of the textile electrodes (ii) after 10 wash cycles. **j** Representative normalized voltage profiles acquired on days 1, 5, and 10 for the three respiratory conditions. **k** Real-time classification results and normalized signal amplitudes recorded before washing and after 10 wash cycles for a subject in the supine position performing normal breathing, inhale-hold, and exhale-hold, with LED indicators displaying the respiratory state predicted by the embedded neural-network model

To reconstruct spatial maps of pulmonary activity, a complete EIT signal-processing pipeline was implemented using the Graz Consensus Reconstruction Algorithm for EIT (GREIT) within the EIDORS MATLAB framework. During each acquisition cycle, low-amplitude alternating currents (< 5 mA peak-to-peak) at 50 kHz were sequentially injected through adjacent electrode pairs, while the resulting voltages were measured across the remaining electrode pairs. For the 16-electrode configuration, current was injected through 16 sequential electrode pairs, and 13 differential voltage measurements were recorded during each injection step, yielding a total of 208 boundary voltage measurements per frame. The resulting dataset exhibited the characteristic U-shaped voltage distribution associated with adjacent-drive EIT systems, with the largest potential amplitudes occurring near the current injection electrodes due to localized impedance gradients (Fig. 7a). These boundary voltages were processed using a finite-element forward model and GREIT inverse reconstruction algorithm to generate two-dimensional maps of relative conductivity changes associated with lung inflation and deflation.

Following successful integration of the LA-MF electrode array and implementation of the reconstruction framework, the wearable system was evaluated on a healthy adult volunteer during controlled respiratory maneuvers. During normal breathing, periodic inhalation and exhalation produced cyclical conductivity changes across the thoracoabdominal region, as illustrated in Fig. S10 and Note 2. Representative images of the breathing condition are shown in Fig. 7b, with the corresponding reconstructed conductivity map in Fig. 7c and normalized voltage profiles in Fig. 7d. The measured signals ranged from approximately 0.12 to 1.0 V and exhibited highly repeatable U-shaped patterns corresponding to a complete EIT measurement cycle.

To evaluate system response under altered respiratory conditions, the subject performed an inhale-hold maneuver immediately following full inspiration. Under this condition, the reconstructed conductivity map (Fig. 7e,f) exhibited minimal temporal variation due to the absence of airflow, and the normalized voltage profile (Fig. 7g) showed a rapid increase during inspiration followed by a stable plateau corresponding to the breath-hold period. The average normalized voltage during this condition was approximately 0.11 V. Together, these results demonstrate that the LA-MF electrode array, combined with the portable EIT system and GREIT reconstruction algorithm, enables reliable discrimination between dynamic and static respiratory states in a fully wearable format.

To further extend the utility of the acquired impedance signals, a machine-learning model was developed to automatically classify respiratory states, including normal breathing, inhale-hold, and exhale-hold. For each EIT frame, the 208 measured voltage values were averaged to generate a representative feature, resulting in a dataset of 600 samples comprising 400 normal breathing cycles, 100 inhale-hold cycles, and 100 exhale-hold cycles. These data were used to train a lightweight neural network implemented in TensorFlow and designed for future deployment on the embedded microcontroller (Fig. S11). The network architecture consisted of two hidden layers with 16 and 8 neurons, each followed by batch normalization and ReLU activation, and a final SoftMax classification layer.

The model was trained using 75% of the dataset (450 samples) with the Adam optimizer and categorical cross-entropy loss. After 50 training epochs, the model achieved approximately 98% training accuracy and greater than 95% validation accuracy (Fig. S12). Evaluation on the remaining test dataset yielded correct classification of 74 out of 91 normal breathing cycles, 23 out of 35 inhale-hold cycles, and 28 out of 35 exhale-hold cycles, corresponding to an overall classification accuracy of approximately 84%. The F1 scores were 0.925 for normal breathing, 0.697 for inhale-hold, and 0.757 for exhale-hold, as summarized in the confusion matrix shown in Fig. 7h. These findings demonstrate that the impedance signals acquired using the LA-MF electrodes contain sufficient information to enable automated respiratory-state recognition in wearable monitoring systems.

Finally, to assess long-term durability during practical use, the electrode-integrated garment was subjected to one machine-wash cycle per day for ten consecutive days. Representative images of the textile electrodes before and after the tenth wash cycle are shown in Fig. 7i(i,ii). No visible degradation, including fraying, delamination, or damage to the conductive regions, was observed after repeated laundering, confirming the mechanical robustness of both the patterned electrodes and the underlying textile structure.

Electrical performance was evaluated by recording normal breathing, inhale-hold, and exhale-hold signals on days 1, 5, and 10. As shown in Fig. 7j, the acquired voltage profiles remained highly consistent across all testing points. The average signal-to-noise ratio (SNR) was 57.5 dB during normal breathing, 56.0 dB during inhale-hold, and 56.5 dB during exhale-hold. After ten washing cycles, the reductions in SNR were limited to only 1.22%, 1.07%, and 1.06%, respectively. These results confirm that the LA-MF electrodes maintain excellent electrical stability and functional reliability under repeated mechanical deformation and laundering, demonstrating their strong potential for long-term wearable respiratory monitoring applications.

Finally, the real-time classification capability of the wearable system was evaluated under practical operating conditions following repeated laundering. During each experiment, the 208 voltage measurements acquired in every EIT cycle were averaged over 2 s intervals and processed directly by the embedded neural network implemented on the microcontroller unit. Continuous monitoring was performed for 150 s using garments before washing (Cycle 0) and after ten machine-washing cycles. As shown in Fig. S13, the LA-MF electrodes retained their structural integrity throughout repeated laundering, with the woven silver yarn bundles preserving their interlaced architecture without visible filament separation, fraying, or damage to the conductive network.

Representative real-time operation of the complete wearable system is shown in Fig. 7k, where the subject wore the garment in a supine position while performing normal breathing, inhale-hold, and exhale-hold maneuvers. During normal respiration, the average normalized potential ranged from approximately 0.20 to 0.30 V, and the embedded model consistently classified the condition as normal, activating the green LED indicator on the portable reader module. During inhale-hold and exhale-hold conditions, the normalized potentials decreased to approximately 0.08–0.10 V and 0.01–0.02 V, respectively. In both cases, the model correctly identified these deviations from the baseline breathing pattern and triggered the red LED indicator, providing immediate visual notification of abnormal respiratory states.

Importantly, identical classification behavior was observed before washing and after ten laundering cycles, confirming that repeated mechanical and environmental stress did not degrade the sensing performance, signal-processing pipeline, or machine-learning functionality of the system. Together, these results demonstrate that the LA-MF electrodes preserve their electrical integrity and sensing fidelity during repeated use and washing, enabling reliable long-term respiratory monitoring in a wearable platform. The integrated garment maintained the natural feel and comfort of a conventional compression shirt, and no skin irritation or user discomfort was observed during extended wear, further highlighting the suitability of this technology for practical continuous health-monitoring applications.

## Conclusions

A selective laser-assisted patterning strategy was developed to transform commercially available MFs into high-performance electronic textiles with circuit-board-like functionality. By exploiting the wavelength-dependent absorption differences between conductive silver yarns and the underlying polyester substrate, a nanosecond-pulsed Nd:YAG laser was used to selectively remove conductive fibers while preserving the structural integrity, flexibility, and breathability of the textile. The process was implemented in a mask-free, chemical-free, and digitally programmable manner, enabling rapid fabrication of fine conductive features (400 µm) directly within the fabric. In addition to defining the desired circuit geometry, the localized thermal energy generated during laser ablation simultaneously fused the exposed ends of adjacent silver yarns, thereby suppressing fraying and enhancing the mechanical robustness of the patterned circuit edges. Systematic optimization identified an operating window of 8–26.5 J·cm^−2^ in which complete removal of the conductive layer was achieved with minimal thermal damage to the supporting textile. The resulting LA-MF exhibited low sheet resistance (approximately 2 mΩ/sq.), preserved tensile properties and breathability, retained more than 94% of its initial conductivity after 10000 bending cycles, and maintained stable electrical performance after more than 20 machine-washing cycles. As a proof of concept, the technology was used to fabricate a garment-integrated 16-electrode EIT array for wearable respiratory monitoring. The resulting platform enabled real-time impedance imaging and machine-learning-based classification of respiratory states, while maintaining consistent performance after repeated laundering. These results demonstrate that selective laser patterning of MFs provides a practical and scalable manufacturing approach that combines the electrical performance of continuous metallic conductors with the comfort, permeability, and durability of conventional textiles. Future works should explore on improving patterning resolution through advanced laser systems with real-time optical alignment, extending the methodology to additional conductive textile materials, integrating multimodal sensing and embedded electronics, and adapting the process to roll-to-roll manufacturing for large-scale production.

## Materials and Methods

### Laser-Assisted Patterning of Metallized Fabric

A commercially available woven MF was procured from EMR Shielding Solutions Inc. (Ontario, Canada) and used as received without further modification. The textile exhibits a dual-layer architecture consisting of a nonconductive base layer composed of tightly woven polyester fibers and a conductive surface layer formed by interwoven pure silver multifilament yarns. The conductive silver yarns are selectively integrated on one side of the textile in both warp and weft directions, forming a periodic grid-like network, as illustrated in Fig. S14a. A three-dimensional isometric representation (Fig. S14b) highlights the characteristic over–under interlacing geometry of adjacent yarns, where alternating fibers are mechanically interlocked in a repeating sequence. This woven configuration provides strong mechanical anchoring between the conductive silver fibers and the underlying polymer matrix, ensuring structural stability while preserving the intrinsic flexibility and breathability of the textile.

At the microscale, each conductive yarn consists of a multi-stranded bundle of pure silver filaments. Individual filaments have an average diameter of approximately 25 µm, with 25 filaments per bundle, resulting in an overall yarn diameter of approximately 140–150 µm (Fig. S15). The silver fibers exhibit electrical performance approaching bulk silver, with a measured sheet resistance of 2 mΩ/sq. The conductive yarns are incorporated at a density of 60 ends per inch (EPI) along the warp direction, while the underlying polyester yarns are spaced at 50 picks per inch (PPI) in the weft direction between conductive traces. The resulting fabric has a thickness of approximately 0.35 mm and an areal density of 150 g·m^−2^. Importantly, the textile maintains high air permeability, measured in the range of 220–250 mm·s^−1^ at a pressure differential of 100 Pa (ASTM D737), highlighting its suitability for wearable applications.

Selective laser patterning of the conductive surface was performed using a nanosecond-pulsed Nd:YAG laser system (PLS6MW, Universal Laser Inc., AZ, USA) operating at a wavelength of 1.06 µm and a repetition frequency of 30 kHz and Q-pulse of 200 (defining the pulse energy). Circuit patterns were digitally designed using CorelDRAW software and transferred to the laser system for raster-based scanning across the metallized surface. During processing, a computer-controlled laser selectively removes conductive silver yarns from predefined regions, thereby defining electrically isolated circuit pathways directly on the textile substrate. Laser power and scanning speed were systematically varied to identify an optimal processing window that enables complete removal of the conductive layer while preserving the integrity of the underlying polyester fibers. Patterning performance was evaluated along both warp and weft orientations to account for anisotropic thermal response and structural heterogeneity associated with the woven architecture.

### Electrical Characterization of Laser-Processed Metallized Fabric

During the laser process optimization, the electrical conductivity of the MF was systematically evaluated as a function of laser power and scanning speed, corresponding to different applied energy densities. Square regions of 1 cm × 1 cm were selectively processed under controlled conditions, and the electrical properties of the modified regions were subsequently characterized. Electrical measurements were performed using a linear four-point probe technique to ensure accurate evaluation of the intrinsic conductivity while minimizing contact resistance effects. In this configuration, four metallic probe tips were aligned sequentially along the laser-patterned conductive pathways, both along the warp and weft directions of the fabric. The probes were connected to a source-measure unit (SMU) (Keithley 2400 SourceMeter, OH, USA), as illustrated in Fig. S16. A constant current (I) was applied through the outer probes, while the voltage difference (ΔV) was measured across the inner probes over a defined segment of the conductive trace. The geometry of the measurement region was characterized by a known probe spacing (*s*), as well as the effective length (*L*) and width (*w*) of the patterned conductive pathway. Based on this configuration, the sheet resistance (${R}_{s}$) was calculated using [77]

1 $${R}_{s}=\frac{\pi }{\mathrm{l}\mathrm{n}(2)}\cdot \frac{\Delta V}{I}=4.53236\cdot \frac{\Delta V}{I}.$$

The measured sheet resistance (Ω/sq.) of the processed regions was used to evaluate electrical performance. In parallel, the surface morphology of the LA-MF was examined at high magnification using optical microscopy (Leica M205 C, Wetzlar, Germany). These observations provided insight into the extent of conductive fiber ablation, structural modification, and potential damage to the underlying textile architecture. Based on the combined electrical and morphological analyses, a well-defined processing window was identified. Within this regime, the laser selectively ablates the conductive silver fibers while preserving the structural integrity of the polyester substrate. This selective removal is critical for enabling high-resolution patterning of conductive pathways while maintaining the mechanical flexibility and textile characteristics of the material.

### Morphological and Elemental Analysis

The surface morphology and elemental composition of the MF were examined before and after laser-assisted ablation at varying energy density levels using a field emission scanning electron microscope (FE-SEM; Helios G4 CX DualBeam, Thermo Fisher Scientific Inc., MA, USA) equipped with an energy-dispersive X-ray spectroscopy (EDS) detector (Oxford Instruments, UK). SEM analysis was performed under high-vacuum conditions with an accelerating voltage of 5–10 kV and a working distance of approximately 5–10 mm, depending on the region of interest. A secondary electron (SE) detector was used to assess surface topography, and backscattered electron (BSE) imaging was employed to enhance contrast between metallic and non-metallic regions. For elemental analysis, EDS measurements were conducted at an accelerating voltage of 15 kV and a probe current of 1.6 nA. Elemental mapping and point analysis were performed on selected ablated and unablated regions to determine the extent of silver removal and to quantify any residual elemental content on the surface. Spectral acquisition time for each point scan was set to 60 s to improve signal-to-noise ratio, and mapping resolution was set at 512 × 400 pixels with a dwell time of 100 µs per pixel.

### Mechanical Strength, Breathability, and Volatile Emission Assessment

To evaluate the effect of laser-assisted patterning on the mechanical integrity of the MF, tensile testing was performed on both pristine and LA-MF samples using a modified strip test adapted from ASTM D5035-11(2024). Fabric specimens were laser-cut into a dog-bone geometry aligned along both the warp and weft directions, with a gauge section of 25 mm × 5 mm to localize deformation within the region of interest. The central portion of the gauge section (20 mm × 5 mm) was selectively laser-ablated under varying energy densities, while the unmodified ends preserved fiber continuity for reliable gripping during testing.

Mechanical characterization was conducted using a universal testing machine (ADMET eXpert 7601, MA, USA) equipped with pneumatic grips and a 50 N load cell. A constant crosshead speed of 2 mm·min⁻^1^ was applied, consistent with ASTM recommendations for woven fabrics, ensuring controlled and reproducible deformation of the hybrid textile–metal structure. Five replicate specimens were tested for each processing condition to ensure statistical reliability. The Young’s modulus was extracted from the slope of the linear elastic region of the resulting stress–strain curves.

To assess the impact of laser processing on fabric breathability, air permeability measurements were conducted using a gas flowmeter system (Textest FX 3300, Switzerland) coupled with a pressure controller, following ASTM D737-18(2023). Circular fabric specimens (1 cm diameter) were laser-ablated under varying energy densities along both warp and weft orientations. A controlled volumetric airflow was applied perpendicular to the fabric surface, and the resulting pressure drop across the sample was measured relative to an open-flow baseline. Airflow resistance (kPa·s·m^−1^) was calculated from the pressure differential at a given flow rate and used as a quantitative indicator of breathability. Five independent measurements were performed for each sample group to evaluate the influence of laser ablation on fabric porosity and airflow characteristics.

To further investigate potential odor-related effects associated with laser processing, the volatile-emission profiles of pristine MF and LA-MF samples were characterized using a multi-sensor chemiresistive gas sensor array. The sensing platform consisted of commercially available gas sensors sensitive to a broad range of volatile compounds, including alcohol (MQ-2), ethanol (MQ-3), methane (MQ-4), NO_x_ (MQ-135), ammonia (MQ-137), and toluene (MQ-138), all sourced from Digi-Key. The sensor array was interfaced with an Arduino MKR WiFi 1010 (Arduino SA, Monza, Italy) for real-time data acquisition.

Measurements were conducted in a controlled enclosed chamber (20 cm × 20 cm × 20 cm), with the sensor array mounted at the top to allow direct exposure to the sample headspace while minimizing external environmental interference. Pristine MF and LA-MF samples (12 cm × 12 cm) were individually placed inside the sealed chamber, and sensor responses were continuously recorded over a 1 h period under identical conditions. In addition, the same measurement was repeated after ten washing cycles to evaluate the stability of the volatile-emission characteristics after repeated laundering as a measure of practical durability during repeated use. This approach enabled comparative analysis of volatile emissions and assessment of any changes in odor-related characteristics induced by laser processing.

### Comparative Reliability Assessment of Laser-Ablated and Printed Conductive Fabrics

Following the identification of optimized laser processing conditions for the selective removal of the metallized layer on MF, the long-term durability and functional stability of the LA-MF samples were systematically evaluated under conditions of mechanical deformation and repeated laundering. For this purpose, conductive traces with dimensions of 100 mm × 2 mm, incorporating contact pads at both ends, were patterned directly onto rectangular MF substrates (150 mm × 10 mm) using the optimized laser parameters. The patterning was achieved through a selective, subtractive laser ablation process that removed the conductive silver fiber layer while preserving the underlying textile structure. The electrical stability of these laser-defined conductive pathways was subsequently monitored as a function of washing cycles to assess durability. For comparative analysis, conductive traces of identical geometry were fabricated on plain woven polyester fabric using a conventional screen-printing approach. A commercially available silver conductive paste (DuPont 5025 silver conductive paste) was deposited through a mesh stencil onto substrates of identical dimensions, followed by thermal curing at 110 °C for 4 h in accordance with the manufacturer’s specifications. These printed samples served as control specimens representative of standard printed textile electronics.

Washability tests for both LA-MF and printed samples were conducted in accordance with ISO 6330, which simulates domestic laundering conditions. In each washing cycle, 200 mL of tap water was mixed with detergent at a concentration of 1:10 (w/v). Samples were subjected to mechanical agitation at 400 r/min for 15 min using a laboratory shaker (IKA KS 3000), followed by air drying under ambient conditions (22 °C, 50% relative humidity). The electrical resistance of each sample was measured after every wash cycle using a four-point probe configuration, with current and voltage probes positioned at the contact pads of the conductive traces to ensure consistent and reproducible measurements of electrical degradation.

Mechanical durability was further assessed through cyclic bending tests designed to simulate repeated deformation during real-world use. Both LA-MF and printed samples were subjected to 10000 bending cycles using a custom-built mechanical testing system that applied a (± 25) mm displacement at a frequency of 0.5 Hz (corresponding to one full bending cycle every 2 s). Electrical resistance was monitored continuously during testing using a data acquisition system (Keysight 34972A Data Acquisition System) to capture real-time changes in conductive performance. To evaluate the combined effects of laundering and mechanical deformation, bending tests were also performed on LA-MF samples after 20 washing cycles under identical testing conditions. This approach enabled direct assessment of post-laundering mechanical reliability and provided insight into the structural and electrical stability of the laser-patterned conductive pathways under coupled environmental and mechanical stress.

### Wearable Electrical Impedance Tomography (EIT) Demonstration

To demonstrate the practical applicability of laser-assisted patterning of MFs for wearable electronics, a fully textile-integrated electrode array was developed for EIT as a proof-of-concept platform for real-time respiratory monitoring. This system highlights the capability of the laser-patterning approach to enable scalable fabrication and seamless integration of electronic functionality into garments for physiological sensing applications. A 16-electrode array was directly patterned onto MF via selective laser removal of the conductive silver fiber layer. The patterned MF was subsequently integrated into a compressive garment designed for close and stable contact with the body. The electrode array was positioned across the anterior abdominal and lower thoracic regions, including both midline and lateral areas, to optimize sensitivity to impedance changes associated with lung expansion and respiratory motion.

Each electrode consisted of a laser-defined conductive pathway incorporating a skin–contact interface and an external connection terminal. Electrical connections to the data acquisition system were established using snap-button connectors integrated at the terminal ends, ensuring stable and repeatable electrical interfacing. To prevent electrical shorting and environmental interference, the conductive interconnects were insulated by overlaying a breathable textile layer, which was laser-patterned with localized openings to expose only the electrode contact regions.

Physiological measurements were performed using a custom-developed portable EIT system designed for noninvasive, radiation-free imaging of internal impedance distributions based on surface voltage measurements. The system was interfaced with the textile electrode array through 16 snap-button connections distributed across the garment.

EIT measurements were conducted using a 16-channel adjacent-drive configuration, enabling spatiotemporal mapping of thoracic impedance. In this approach, low-amplitude alternating currents (0.4–1 mA) at a frequency of 50 kHz were sequentially injected through adjacent electrode pairs. This frequency range was selected to ensure sufficient tissue penetration while minimizing electrode polarization effects. For each current injection step, differential voltages typically in the millivolt to volt range were measured across the remaining electrode pairs, generating a complete set of boundary voltage data for each acquisition frame. The measurement sequence cycled through all electrode combinations (16 injection steps per frame), enabling full tomographic reconstruction.

The acquired boundary voltage data were processed using finite-element-based forward models and inverse reconstruction algorithms, including regularized Gauss–Newton and GREIT methods, to generate cross-sectional two-dimensional conductivity maps. Temporal variations in the reconstructed impedance distributions were correlated with changes in pulmonary air volume, enabling visualization of lung expansion, regional ventilation patterns, and dynamic respiratory behavior.

The portable EIT system comprised a waveform generator (AD9833, Analog Devices, MA, USA), a high-pass filtering stage, an instrumentation amplifier, a voltage-controlled current source (VCCS), a multiplexer/demultiplexer, an AC/DC conversion stage, an analog-to-digital converter (ADS1115, Texas Instruments, TX, USA), and a microcontroller (ESP32-S2, Digi-Key, MN, USA) (Fig. S17). The AD9833 generated a stable 50 kHz sinusoidal waveform (0.6 V peak-to-peak, 0.21 V RMS), which was conditioned through an RLC high-pass filter to remove low-frequency noise and DC offsets. The signal was then converted into a controlled current output (0.4–1 mA) using a modified Howland current pump (Fig. S18).

Electrode selection and signal routing were managed through a multiplexing system controlled by the ESP32-S2, enabling automated switching between current injection and voltage measurement pairs. The resulting voltage signals were amplified using an instrumentation amplifier to enhance signal fidelity and subsequently digitized using the analog-to-digital converter (ADC) for high-resolution data acquisition. The microcontroller coordinated waveform generation, electrode switching, timing synchronization, and data communication with an external computing system for real-time visualization and image reconstruction.

All EIT measurements were conducted on healthy adult volunteers wearing the compressive garment integrated with LA-MF electrodes. The study protocol included two physiological conditions: (i) a resting baseline and (ii) a post-exertion state. For baseline measurements, subjects remained seated in an upright position for 10 min under ambient conditions, while continuous EIT data were recorded to establish steady-state impedance profiles. To induce physiological variation, subjects performed a structured high-intensity exercise protocol consisting of jumping jacks, squats, and burpees in 1 min intervals over a total duration of 10 min. Immediately following exercise, EIT measurements were repeated while maintaining the same seated posture. This protocol induced measurable thoracoabdominal impedance variations associated with increased respiratory effort and muscular activity. To evaluate system performance across varying body compositions, the same experimental procedure was conducted on a second participant with a higher body mass index (BMI). This comparative assessment provided additional validation of the robustness and adaptability of the textile-based EIT platform under different physiological conditions.

### Ethical Compliance

All human subject procedures were performed with informed consent in accordance with institutional ethical guidelines and the principles outlined in the Declaration of Helsinki.

## Supplementary Information

Below is the link to the electronic supplementary material.

Supplementary file1 (DOCX 46496 KB)

## Data Availability

The data supporting the findings of this study are available from the corresponding author upon reasonable request.
