# Supplementary material for: Laser-Assisted Patterning of Metallized Fabrics for Washable and Durable Electronic Textiles
Source: Adv Fiber Mater. 2026 Aug 24;8(6):2552–77. doi: 10.1007/s42765-026-00761-8 (PMC13739953; doi:10.1007/s42765-026-00761-8)
Supplement: Supplementary file 1 [file 42765_2026_761_MOESM1_ESM.docx]

Supporting Information

**Laser-Assisted Patterning of Metallized Fabrics for Washable and Durable Electronic Textiles**

Muhammad Masud Rana^1,2,#^, Sotoudeh Sedaghat^2,3,#^, Akshay Krishnakumar^1,2^, Devendra Sarnaik^2,3^, Praveen Srinivasan^1,2^, Yashwanth Ramesh^1,2^, Md Mahabubur Rahman^1,2^, Rahim Rahimi^1,2,3,*^

*^1^School of Electrical and Computer Engineering, Purdue University, West Lafayette, IN 47907, USA*

*^2^Birck Nanotechnology Center, Purdue University, West Lafayette, IN 47907, USA*

*^3^School of Materials Engineering, Purdue University, West Lafayette, IN 47907, USA*

*^#^Authors contributed equally to the manuscript*

*^*^Corresponding author*

*Email:* [*rrahimi@purdue.edu*](mailto:rrahimi@purdue.edu)

**Fig. S1** Mapping of electrical sheet resistance of laser-ablated metallized fabric (LA-MF) as a function of laser power and scanning speed for patterning performed along the **a** warp and **b** weft directions. The plots illustrate the transition from conductive to insulating behavior across the investigated laser processing conditions and define the selective-ablation operating window for effective removal of conductive silver fibers.

**Fig. S2** Reproducibility assessment of laser-ablated metallized fabric (LA-MF). **a–d** Representative images of four independently processed MF samples fabricated under identical laser processing conditions. **e** Corresponding F-statistical analysis of sheet resistance measurements across all patterned regions, demonstrating minimal inter-sample variability and high reproducibility of the laser ablation process.

**Fig. S3.** Evaluation of selective laser ablation under out-of-plane fabric surface variations. **a** Schematic illustration of the experimental setup, where the metallized fabric (MF) was mounted at controlled inclination angles to introduce progressive focal-height variation during laser processing. **b–f** Representative optical images of laser-ablated regions processed under increasing fabric inclinations, showing the effect of defocusing on ablation uniformity and conductive-fiber removal. **g** Sheet resistance as a function of out-of-focus distance relative to the laser focal plane, identifying three distinct processing regimes corresponding to complete ablation, partial ablation, and insufficient ablation of the conductive silver layer.

**Fig. S4** Resolution characterization of selective laser patterning on metallized fabric (MF). **a** Optical microscopy images of pristine MF before and after selective laser ablation. **b** High-magnification image of the pristine woven silver-fiber network prior to processing. Laser-patterned conductive traces with widths of **c** (400 ± 20) µm, **d** (750 ± 20) µm, and **e** (1200 ± 50) µm, demonstrating high-resolution subtractive patterning through selective removal of conductive silver fibers surrounding the defined circuit traces.

**Fig. S5** Characterization of laser-ablated conductive pathways on metallized fabric (MF) fabricated through selective subtractive laser ablation along the warp and weft directions. **a** Optical images of conductive traces patterned along the warp direction with widths of 0.4, 0.6, 0.8, 0.9, and 1.0 mm. **b** Corresponding conductive traces patterned along the weft direction with identical dimensions. All traces were fabricated with a constant length of 1 cm. **c** Sheet resistance of the laser-defined conductive pathways as a function of trace width for both processing orientations. Error bars represent standard deviation from five independent measurements (n = 5).

**Fig. S6** Schematic comparison of representative e-textile fabrication strategies for integrating conductive circuitry into textile substrates, including screen printing, conductive-thread embroidery, flexible PCB integration, laser cutting with conductive-fabric lamination, and selective laser ablation of metallized fabrics (this work). The illustration highlights the relative process complexity, number of fabrication steps, and conductive-pathway integration approaches associated with each manufacturing method.

**Supporting Note 1:**

To evaluate the change in flexibility of the textile upon laser ablation, the pristine metallized fabric (MF) and the fabric processed under the optimized laser condition were characterized by using a standard drape test based on the projected-area method in accordance with ISO 9073-9 method B (Fig. S7a-b). For this test, circular specimens with a diameter of 300 mm were placed on a 180 mm diameter support disc, and the measurements were performed at 0°, 45°, and 90° fabric orientations to account for possible directional effects associated with the woven structure. For each orientation, the draped specimen was imaged using a camera positioned at a fixed distance of 60 cm above the sample. The captured images were then processed in ImageJ to extract the projected silhouette and determine the corresponding projected area for drape coefficient calculation.

As observed, the draped specimens of the pristine MF exhibit smooth and continuous deformation profiles across the investigated orientations, with the projected silhouettes reflecting the natural draping behavior of the woven textile structure (Fig. S7c). Across the tested orientations of 0°, 45°, and 90°, the draped profiles remain consistent, forming symmetric folds around the support disc while maintaining similar projected contour shapes, indicating uniform draping behavior of the pristine fabric irrespective of orientation. Similarly, the laser-ablated metallized fabric (LA-MF) at optimized laser energy density exhibits comparable draping behavior across the investigated orientations, as shown in Fig. S7c. At 0°, 45°, and 90° orientations, the draped specimens form smooth folds around the support disc with projected silhouettes that closely resemble those observed for the pristine MF.

Subsequently, the drape coefficient was calculated from the extracted projected areas obtained from the processed silhouette images for each fabric orientation for both the pristine MF and the LA-MF to enable direct comparison of their draping behavior. The drape coefficient ($DC$) was calculated as

$DC(\%)=\frac{A_{d}-A_{s}}{A_{D}-A_{s}}\times100$	(1)

where $A_{d}$ is the projected area of the draped specimen, $A_{s}$ is the area of the support disc, and $A_{D}$ is the original area of the undeformed circular specimen.

As observed from Fig. S7d, the pristine MF exhibited drape coefficients of approximately 57.3%, 56.1%, and 57.8% at 0°, 45°, and 90°, respectively, corresponding to an average drape coefficient of 57.1%. Under the same conditions, the LA-MF at optimized laser energy density exhibited drape coefficients of approximately 63.4%, 64.9%, and 63.7% at 0°, 45°, and 90°, respectively, corresponding to an average drape coefficient of 64.0%.

These results indicate that laser processing produced only a moderate change in drape behavior, while the overall projected profiles remained comparable across all tested orientations. This observation is consistent with textile mechanics literature, where fabric drape is governed by the combined effects of bending rigidity, shear resistance, areal mass distribution, and yarn architecture. Thus, the proposed laser-assisted ablation process is spatially confined to the MF surface region and does not introduce extensive structural modification throughout the fabric body.

**Fig. S7** Drape behavior characterization of pristine metallized fabric and laser-ablated metallized fabric. **a** Circular specimen of pristine metallized fabric used for the drape test. **b** Circular specimen of metallized fabric processed under the optimized laser ablation condition. **c** Representative drape test images showing raw draped specimens and the corresponding projected silhouettes obtained from image processing at fabric orientations of 0°, 45°, and 90° for both pristine and laser ablated fabrics. **d** Drape coefficient calculated from the projected area measurements for the tested fabric orientations. All measurements were performed with n = 5 independent samples.

**Fig. S8** Volatile-emission characterization of pristine metallized fabric (MF) and laser-ablated metallized fabric (LA-MF) before and after washing. Representative photographs of the VOC sensing setup showing the sealed chamber with the integrated gas sensor array for **a** pristine MF and **b** LA-MF. Time-dependent gas concentration profiles relative to ambient-air conditions for **c** pristine MF and **d** LA-MF before and after 10 washing cycles. **e** Average change in measured gas concentration after 1 h for pristine MF and LA-MF under all tested conditions, demonstrating negligible VOC generation following laser processing and washing cycles.

**Fig. S9** **a** Geometrical layout and dimensional schematic of the 16-electrode array patterned on metallized fabric for electrical impedance tomography (EIT) based physiological lung activity monitoring. The layout specifies the overall electrode length and width, trace width and routing length, connector diameter. **b** Photograph of the selectively laser-ablated metallized fabric showing electrically isolated interconnect traces and electrode pads defined with high spatial resolution.

**Supporting Note 2:**

Electrical conductivity in biological tissues arises primarily from ionic transport through extracellular and intracellular fluids. In the thoracic region, the effective bulk conductivity therefore depends on the relative contributions of highly conductive tissues such as blood, muscle, and lung parenchyma together with the presence of air within the pulmonary alveoli. As illustrated in Fig. S10, during inhalation the lungs expand and the volume fraction of air within the alveolar structures increases. This expansion increases the proportion of air within the lung cavities relative to the surrounding conductive tissues and fluids. Since air exhibits extremely low electrical conductivity compared with biological tissues and blood, the increase in alveolar air volume reduces the effective electrical conductivity of the lung region and increases the measured electrical impedance of the thoracic domain. Conversely, during exhalation the air volume within the lungs decreases, and the relative contribution of conductive tissue and fluid correspondingly increases. This leads to an increase in the effective conductivity of the thoracic region and a decrease in the measured impedance. These conductivity variations correspond to the inhalation and exhalation conditions depicted in Fig. S10, arising from the dynamic changes in pulmonary air content, tissue density, and fluid distribution within the lungs during the respiratory cycle.

**Fig. S10** Schematic illustration of conductivity variation in the thoracic region during inhalation and exhalation detected using a circumferential electrical impedance tomography (EIT) electrode array. The blue shaded regions represent the conductivity distribution within the lungs, where lighter regions indicate lower conductivity associated with higher air content and darker regions indicate relatively higher conductivity corresponding to increased tissue and fluid contribution.

**Fig. S12** Training and validation performance of the fully connected neural network. **a** Evolution of training and validation loss as a function of epoch. **b** Corresponding training and validation accuracy over the training period, demonstrating stable convergence with minimal divergence between training and validation curves.

**Fig. S11** Schematic of the neural network architecture used for respiratory phase classification. The network consists of an input layer followed by two fully connected hidden layers with 16 and 8 neurons, respectively, each incorporating batch normalization and ReLU activation. A final softmax layer outputs class probabilities corresponding to exhalation hold, inhalation hold, and normal breathing.

**Fig. S13** Structural stability of laser-ablated metallized fabric (LA-MF) electrodes after repeated washing cycles. Photograph of the LA-MF electrode integrated garment and corresponding optical microscopy images of the conductive textile structure after **a** 1, **b** 5, and **c** 10 washing cycles.

**Fig. S14**. Schematic illustration of the woven metallic textile architecture showing **a** top view of the warp–weft interlaced structure and **b** three-dimensional perspective highlighting the over–under yarn arrangement.

**Fig. S15** Optical microscopy images of conductive silver (Ag) fibers. **a** Pristine conductive Ag yarn composed of multiple intertwined filaments. **b** Individual filaments isolated from the yarn bundle, revealing filament-level morphology **c,d** Single conductive filaments imaged at higher magnification, showing filament continuity and diameter uniformity.

**Fig. S16** Schematic illustration of the four-probe electrical measurement configuration used to determine the conductance of the laser processed MFs, showing probe placement along the conductive pathway and the current injection and voltage sensing arrangement.

**Fig. S17** Block diagram of the portable electrical impedance tomography (EIT) module. The system integrates a programmable signal generator, multiplexing network, analog front-end, and analog-to-digital converter (ADC) with an ESP32-S2 microcontroller, enabling controlled current injection, differential voltage sensing, and on-board data recording with embedded machine-learning–based decision making for real-time processing and classification.

**Fig. S18** Howland current pump used for controlled current injection. Schematic of the enhanced Howland current source employed to generate a stable, load-independent AC excitation current. [1]

**Modified Howland current-pump equation:**

$I_{Load}=\frac{A_{Howland}\times(V_{p}-V_{n})}{R_{5}}$	(2)

Where Howland gain was calculated by the resistors $R_{1}$, $R_{2}$, $R_{3}$ and $R_{4}$:

$A_{Howland}= \frac{R_{2}}{R_{1}}; (R_{1}=R_{3} and R_{2}=R_{4})$	(3)

**Table S1 Comprehensive Comparison of Wearable E-Textile Circuit Fabrication Methods**

| Fabrication Method | Conductive Material | Resistivity/ Conductivity | Resolution (mm) | | Feature Size (mm) | Durability | Washability (cycles) | Processing Time | Substrate | Process Complexity | Application | Limitations | Ref. |
| --- | --- | --- | --- | --- | --- | --- | --- | --- | --- | --- | --- | --- | --- |
| Screen Printing | Cu complex Ink | 9.7 ×10^6^ S/m | | - | 1.3 track width | 1000 stretching cycles | 2 | >1 h | Nylon and Polyester | Low | Wearable gloves | Copper oxidation | [2] |
|  | Silver (Ag) Ink | 1.64 Ω/sq. | | ≥1 | 20–30 | 30% strain | 10 | 0.5 h | Knitted cotton fabric | Low | ECG^#^ Electrode | High sheet resistance | [3] |
|  | Graphene Ink | (96 ± 8) Ω/sq. | | - | 20 × 15 | 2000 bending cycles | 9 | 2 h | Cotton fabric | Low | Vital-sign monitoring | Lower conductivity than Ag inks | [4] |
|  | PEDOT:PSS^$^ | (0.09–0.48) kΩ | | - | - | 20,000 Martindale cycles | 20 | 24 h | Cotton and Polyester | Medium | LED^%^ circuits | Lower conductivity without binder | [5] |
| Inkjet Printing | Graphene-Silver Composite Ink | (2.11–30.9) Ω/sq. | | 0.0215 | 10 × 10 | 1000 bending cycles | - | 1 h | Cotton woven fabric | Medium | Wearable strain sensor | Ink cost, nozzle clogging | [6] |
|  | Copper (Cu) Nanoparticle Ink | (0.3–0.5) Ω/sq. | | 2 | >2 | Moderate; oxidation-prone | 5 | >30 min | Polyester woven textile | High | Conductive interconnects on fabrics | Poor wash durability | [7] |
|  | PEDOT: PSS^$^ | (1.6–7.5) Ω/sq. | | 0.05–0.1 | 1-5 | Very flexible | 3 | >1 h | Nonwoven PET^*^ (polyester) | Low | Soft, breathable electrode | Low conductivity | [8] |
| Embroidery (Conductive Thread) | Silver-plated Nylon thread | - | | - | 50×30 | Good without bleach | <30 | - | Cotton backing Jersey | Low | Biopotential sensing | Lower conductivity | [9] |
|  | Stainless Steel thread | 91.5 Ω/m | | 0.2–0.5 (thread size) | 2–10 (stitch length) | 1000 bending cycles | 5 | Slower stitching rate | Knitted & woven textiles | Medium | ECG^#^, strain sensors | Low flexibility, stiffer fabric | [10] |
|  | CNT** /WPU^##^ composite yarns | 3×10^3^ S/m | | - | 0.27 (yarn dia) | Good mechanical stability | 10 | (6~12) min per pattern | Polyester | Medium | Wearable interconnects | Lower conductivity | [11] |
|  | MXene coated cotton yarn | 5.1 Ω | | - | - | 500 bending cycles | 5 | 30 h | Nylon–spandex fabric | High | EMI^$$^ Shielding | No patterned resolution control | [12] |
| Flexible PCB Attachment | Copper-Polyimide (PI) | 16.5 Ω/m | | 0.1 | 0.15 track width | 100 stretching cycles | ≥25 | 1 min | Knitted fabric | High | Cu–PI circuits | Rigid zones, breathability | [13] |
|  | Silver (Ag) ink on PET | (2–5×10^6^) S/m | | - | 0.1–0.3 | - | - | (20–30) min | PET^*^ | High | Thin-film circuit wiring | Not stretchable | [14] |
| Lithography | Nickel (Ni) | <18 Ω/cm | | 0.10–0.20 | 0.08–0.10 | 10,000 bending cycles | 20 | (5~6) h | Cotton-polyester with adhesion promoter | High | Biosensing | Etching and multi-mask steps | [15] |
|  | Silver nanowires (AgNWs) | (7–20) Ω/sq. | | 0.2 | 0.2-0.5 | 160 bending cycles | - | Fast | Hydrogels, PDMS^@^ | High | Soft bioelectrodes, pressure sensors | Non-textile substrate | [16] |
| Laser-Based | Laser-induced graphene (LIG) on Kevlar | 10.6 Ω | | - | 25×30 | - | - | 2.5 min | Kevlar textile (aramid) | Low | Zn–air batteries, ECG^#^ electrodes | Moderate conductivity, non-solderable | [17] |
|  | Ni–Cu metallized polyester woven fabric | (0.05–0.08) Ω/sq. | | - | >0.4 | >20% strain | - | Fast | Polyester textile | Medium | NFC^$$^ tag | Limited resolution | [18] |
|  | **Silver (Ag) woven fabric** | **2 mΩ/sq.** | | **0.1** | **0.4** | **10,000 bending cycles** | **>20** | **Fast** | **Polyester** | **Low** | **Wearable electrode** | **Subtractive, restricted to certain MF**^%%^ | **Our work** |

^#^Electrocardiogram, ^$^Poly(3,4-ethylenedioxythiophene) polystyrene sulfonate, ^%^Light-emitting diode, ^*^Polyethylene terephthalate, **Carbon nanotubes, ^##^Waterborne polyurethane, ^$$^Electromagnetic interference, ^@^Polydimethylsiloxane, ^$$^Near field communication, ^%%^Metallized fabric

**References**

1. Lam IV. Analysis of improved Howland current pump configurations. Texas Instruments; 2023. [www.ti.com](http://www.ti.com)

2. Farraj Y, Kanner A, Magdassi S. E-textile by printing an all-through penetrating copper complex ink. ACS Appl Mater Interfaces. 2023; 15: 21651. https://doi.org/10.1021/acsami.3c02242

3. Nigusse AB, Malengier B, Mengistie DA, Tseghai GB, Van Langenhove L. Development of washable silver printed textile electrodes for long-term ECG monitoring. Sensors. 2020; 20: 6233. https://doi.org/10.3390/s20216233

4. Xu X, Luo M, He P, Yang J. Washable and flexible screen printed graphene electrode on textiles for wearable healthcare monitoring. J Phys D Appl Phys. 2020; 53: 125402. https://doi.org/10.1088/1361-6463/ab5f4a

5. Ojstršek A, Gorgieva S. Tailoring of durable conductive and UV-shielding properties on cotton and polyester fabrics by PEDOT:PSS screen-printing. Polymers. 2020; 12: 2356. https://doi.org/10.3390/polym12102356

6. Karim N, Afroj S, Tan S, Novoselov KS, Yeates SG. All inkjet-printed graphene-silver composite ink on textiles for highly conductive wearable electronics applications. Sci Rep. 2019; 9: 8035. https://doi.org/10.1038/s41598-019-44420-y

7. Azar GTP, Danilova S, Krishnan L, Fedutik Y, Cobley AJ. Selective electroless copper plating of ink-jet printed textiles using a copper-silver nanoparticle catalyst. Polymers. 2022; 14: 3467. https://doi.org/10.3390/polym14173467

8. Guo Y, Otley MT, Li M, Zhang X, Sinha SK, Treich GM, Sotzing GA. PEDOT:PSS “wires” printed on textile for wearable electronics. ACS Appl Mater Interfaces. 2016; 8: 26998. https://doi.org/10.1021/acsami.6b08036

9. Gaubert V, Gidik H, Bodart N, Koncar V. Investigating the impact of washing cycles on silver-plated textile electrodes: a complete study. Sensors. 2020; 20: 1739. https://doi.org/10.3390/s20061739

10. Sofronova D, Angelova RA. Embroidery of conductive threads in textiles. In: Sewing machines and processes. Boca Raton: CRC Press; 2025. p. 228. https://doi.org/10.1201/9781003293071-12

11. Chen W, Cai M, Wu J, Ma H, Liu W, Xu F. Highly conductive, durable, washable, and scalable composite yarn for multifunctional wearable electronic applications. Compos Sci Technol. 2023; 241: 110115. https://doi.org/10.1016/j.compscitech.2023.110115

12. Peng J, Cheng H, Liu J, Han W, Wu T, Yin Y, et al. Superhydrophobic MXene-based fabric with electromagnetic interference shielding and thermal management ability for flexible sensors. Adv Fiber Mater. 2023; 5: 2099. https://doi.org/10.1007/s42765-023-00328-x

13. Veske P, Bossuyt F, Vanfleteren J. Testing for wearability and reliability of TPU lamination method in e-textiles. Sensors. 2021; 22: 156. https://doi.org/10.3390/s22010156

14. Arrese J, Vescio G, Xuriguera E, Medina-Rodriguez B, Cornet A, Cirera A. Flexible hybrid circuit fully inkjet-printed: surface mount devices assembled by silver nanoparticles-based inkjet ink. J Appl Phys. 2017; 121: 104904. https://doi.org/10.1063/1.4977961

15. Wang P, Ma X, Lin Z, Chen F, Chen Z, Hu H, et al. Well-defined in-textile photolithography towards permeable textile electronics. Nat Commun. 2024; 15: 887. https://doi.org/10.1038/s41467-024-45287-y

16. Ko Y, Kim J, Kim D, Yamauchi Y, Kim JH, You J. A simple silver nanowire patterning method based on poly(ethylene glycol) photolithography and its application for soft electronics. Sci Rep. 2017; 7: 2282. https://doi.org/10.1038/s41598-017-02511-8

17. Wang H, Wang H, Wang Y, Su X, Wang C, Zhang M, et al. Laser writing of Janus graphene/Kevlar textile for intelligent protective clothing. ACS Nano. 2020; 14: 3219. https://doi.org/10.1021/acsnano.9b08638

18. Gaubert V, Boddaert X, Djenizian T, Delattre R. Textile electronic circuits from laser-patterned conductive fabric. Adv Eng Mater. 2023; 25: 2201548. https://doi.org/10.1002/adem.202201548
